# Lectin-Coated Silver/Silver Chloride Nanoparticles in Combination with Gentamicin: A Strategy to Preserve Antibiotic Efficacy at Lower Doses Against Pathogenic Planktonic Bacteria

**DOI:** 10.1021/acsomega.5c03081

**Published:** 2025-11-24

**Authors:** Viviane Brito Andrade, Diógenes G. da S. Fernandes, Dnane Vieira Almeida, Geomar F. Cruz, Tamara Jarosi Handajevsky, Daiany A. Ribeiro, Claudener S. Teixeira, André Luis Coelho da Silva, Fernanda Dias da Silva, Wanius Garcia

**Affiliations:** † Centro de Ciências Naturais e Humanas, 74362 Universidade Federal do ABC (UFABC), Santo André, São Paulo 09280-560, Brazil; ‡ Centro de Ciências Naturais e Humanas, 74362 Universidade Federal do ABC (UFABC), São Bernardo do Campo, São Paulo 09280-560, Brazil; § Centro de Ciências Agrárias e da Biodiversidade, 423875 Universidade Federal do Cariri (UFCA), Crato, Ceará 63048-080, Brazil; ∥ Laboratório de Biotecnologia Molecular (LabBMol), Departamento de Bioquímica e Biologia Molecular, 28121 Universidade Federal do Ceará, Fortaleza, Ceará 60440-970, Brazil

## Abstract

The rampant overuse of antibiotics in recent decades has significantly contributed to the emergence of antibiotic resistance. This highlights the need for new antibacterial strategies to reduce dependence on traditional antibiotics and slow the development of resistance. Concanavalin A-coated silver/silver chloride nanoparticles (ConA/Ag/AgCl-NPs) offer a promising alternative, combining the antimicrobial activity of metallic silver with the ability of concanavalin A to interact with both membrane carbohydrates and antibiotic gentamicin. The aim of this study is to investigate the synergistic antibacterial activity of gentamicin combined with ConA/Ag/AgCl-NPs against pathogenic bacteria. Our results confirmed that gentamicin interacts with ConA/Ag/AgCl-NPs, altering their plasmonic resonance properties. The combination of gentamicin with ConA/Ag/AgCl-NPs significantly reduced the minimum inhibitory concentration (MIC) values of both, from 0.19 μg/mL and 0.04 μg/mL to 0.02 μg/mL and 0.01 μg/mL, respectively, required to inhibit planktonic *Staphylococcus aureus*. Similarly, the MIC values required to inhibit planktonic *Pseudomonas aeruginosa* reduced from 0.39 μg/mL and 0.02 μg/mL to 0.02 μg/mL and 0.0012 μg/mL, respectively. In both cases, a reduction of more than 90% in the amount of gentamicin used was observed. These data demonstrate synergistic antibacterial activities, as revealed by the fractional inhibitory concentration indexes below 0.5. Additionally, a killing kinetic assay confirmed bactericidal effects of the combination against both strains tested. Conversely, for both strains, ConA/Ag/AgCl-NPs exhibited substantial reduction in biofilm formation only at a concentration at least 500-fold greater than the MIC values observed for planktonic cells. Therefore, the strategy presented here allows for significantly lower doses of gentamicin to achieve the same level of effectiveness against pathogenic planktonic bacteria.

## Graphical abstract

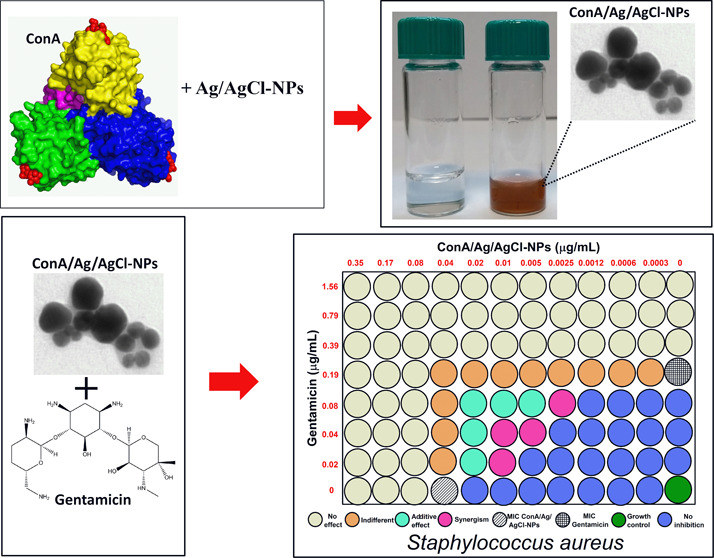

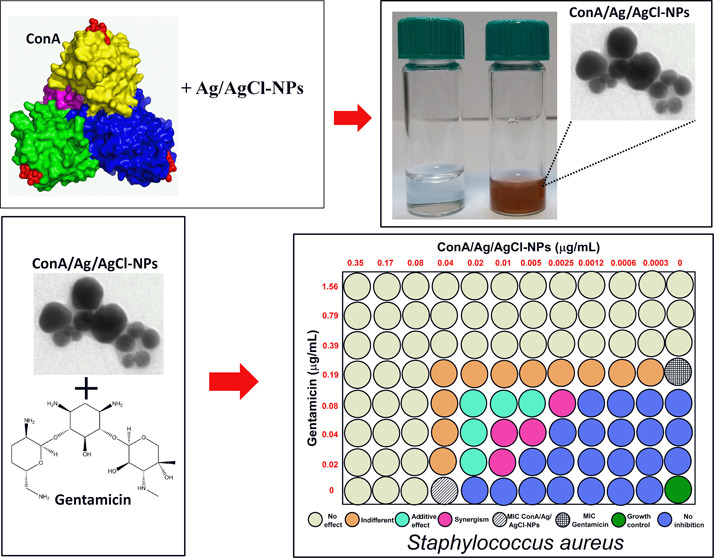

## 1 Introduction

Lectins, proteins known for their reversible binding to specific sugars, also exhibit antimicrobial properties.^1,2^ The first legume lectin reported was the Concanavalin A (ConA) isolated from the seeds of the jack bean (*Canavalia ensiformis*).^3^ ConA specifically targets and binds to mannose or glucose sugars on the cell surface through its carbohydrate recognition domain (CRD).^4^ A recent study demonstrated that gentamicin, an aminoglycoside antibiotic that inhibits bacterial protein synthesis, exhibits enhanced antibacterial activity against multidrug-resistant (MDR) *Staphylococcus aureus* and *Escherichia coli* when combined with ConA. However, no significant antibacterial activity was observed against MDR *Pseudomonas aeruginosa* with the same combination. The study also revealed an interaction between gentamicin and the lectin’s CRD.^5^

Several types of nanoparticles (NPs), including metal oxides, silver halides, and mesoporous silica NPs,^6–11^ possess intrinsic antibacterial properties. Additionally, they can serve as nanocarriers to deliver antibiotics or other therapeutic agents. The potential of silver-based NPs in biomedical applications stems from their multifaceted approach to combating bacteria. These NPs exhibit direct antibacterial properties, low cytotoxicity toward mammalian cells, and a reduced propensity to induce resistance in bacteria. The mechanisms by which silver-based NPs exert their antibacterial effects are complex and not fully understood, but several key pathways have been identified. First, in their colloidal suspension form, silver-based NPs can interact and compromise bacterial membrane integrity, causing cell lysis and the leakage of intracellular materials. Second, silver ions released from the NPs can disrupt essential bacterial functions by interacting with proteins and other internal structures.^12^ Third, silver-based NPs can generate reactive oxygen species (ROS) within the bacterial cell. These ROS are highly reactive molecules that can damage cellular components, leading to oxidative stress and cell death.^13^

The alarming rise of bacterial resistance to antibiotics, leading to an estimated 700,000 deaths annually from MDR bacteria,^14–16^ has become a serious global problem. Additionally, a misconception persists that existing antibiotics can still adequately address this public health threat.^17,18^ One potential solution to combat this challenge lies in nanomaterials with antibacterial properties. Silver-based NPs functionalized with proteins and/or antibiotics are a promising approach. This synergistic combination can enhance antibiotic efficacy, leading to faster bactericidal activity and potentially reducing the emergence of resistant bacteria. Furthermore, these functionalized NPs may offer additional antibacterial activity by disrupting biofilm formation in bacteria.^12,18^

Functionalizing the surfaces of NPs with proteins and/or antibiotics emerges as a promising strategy to enhance their biological response.^19,20^ Our group recently demonstrated that ConA-coated silver/silver chloride NPs (ConA/Ag/AgCl-NPs) retain their ability to agglutinate rabbit erythrocytes. This indicates that the CRD of ConA remains functional on the NPs surface, enabling interaction with biological membrane carbohydrates and potentially even with antibiotics.^21^ Notably, ConA and ConA-like lectins have been shown to interact with aminoglycosides, potentially modulating antibiotic activity against MDR bacteria.^5,22^

In this context, the alarming rise of antibiotic resistance necessitates the development of novel therapeutic strategies to lessen our reliance on existing antibiotics, preserve their effectiveness, and minimize the emergence of resistant strains. ConA/Ag/AgCl-NPs hold promise as a novel alternative for aminoglycoside antibiotic delivery (e.g., gentamicin) due to their multifaceted properties. These NPs possess intrinsic antibacterial activity, low cytotoxicity toward mammalian cells (AgCl is less toxic to mammalian cells compared to Ag),^23^ and good biocompatibility, potentially offering several advantages over traditional antibiotics.

ConA binds to mannose and glucose, key sugar components of bacterial outer membrane lipopolysaccharides and peptidoglycans. By conjugating ConA to Ag/AgCl-NPs, we hypothesized that the lectin would facilitate the nanoparticles’ close interaction with bacterial cell surfaces. This interaction would aim to increase the local concentration of Ag/AgCl-NPs at the site of action, potentially improving their disruptive effects on the bacterial cell wall/membrane and thus enhancing overall antibacterial efficacy, including the synergistic effect in combination with gentamicin. This study investigates the potential of ConA/Ag/AgCl-NPs as a synergistic strategy to enhance gentamicin efficacy, potentially reducing antibiotic dependence and treatment duration. We explore the combined effect of gentamicin and ConA/Ag/AgCl-NPs against the pathogenic bacteria *S. aureus* and *P. aeruginosa*.

## 2 Materials and Methods

### 2.1 Purification of ConA Lectin from Seeds of *C. ensiformis*

ConA lectin was purified as described previously.^5^ Briefly, seeds from *C. ensiformis* were milled to a fine powder. Subsequently, 5 g of powder were incubated in 50 mL of 150 mM NaCl solution under continuous stirring at 25 °C for 4 h. Then, the solubilized proteins were separated by centrifugation at 10,000*g* for 20 min at 4 °C. ConA was then purified by affinity chromatography using a Sephadex-G50 column (Sigma, Saint Louis, USA) equilibrated with 100 mM NaCl. The unbound proteins were washed out with the same solution, and ConA was eluted from the column using 0.1 M glycine at pH 2.6. The collected fractions containing ConA were analyzed by sodium dodecyl sulfate–polyacrylamide gel electrophoresis (SDS–PAGE). The concentration of ConA was determined by absorbance at 280 nm using a theoretical extinction coefficient based on the amino acid composition.^24^

### 2.2 Synthesis of ConA-Coated Silver/Silver Chloride Nanoparticles

Synthesis of ConA-coated silver/silver chloride NPs (ConA/Ag/AgCl-NPs) was performed using a previously reported methodology with slight modifications.^21^ All reagents were purchased from Sigma-Aldrich. Briefly, 5 mL of 1 mM Tris-HCl buffer (pH 8.0) containing 1 mM AgNO_3_ and 0.1 mg/mL of ConA was irradiated with visible light for 30 min without stirring at 25 °C. Controls were also performed under irradiation in the absence of ConA and AgNO_3_. The ConA/Ag/AgCl-NPs were then washed with ultrapure water by centrifugation at 4000*g* for 4 min. Additionally, Ag/AgCl-NPs coated by nonantimicrobial plant extract from *Stryphnodendron adstringens* (*Sa*/Ag/AgCl-NPs) were produced and purified using the same methodology.^21,25^

### 2.3 Characterization of ConA/Ag/AgCl-NPs

A double-beam UV–vis spectrophotometer (Biachrom Libra) was used to measure the absorbance spectrum of the ConA/Ag/AgCl-NPs in ultrapure water, in the absence and presence of gentamicin, over a wavelength range of 300–800 nm. Dynamic light scattering (DLS) and electrophoretic light scattering (ELS) measurements were performed at 25 °C using a ZetaSizer Nano ZS. ConA/Ag/AgCl-NPs samples, suspended in ultrapure water, were loaded into 10 mm diameter cuvettes. ConA and ConA/Ag/AgCl-NPs were subjected to Fourier transform infrared spectroscopy (FTIR) measurements. The measurements were carried out on a PerkinElmer instrument in transmittance mode between 700 and 4000 cm^–1^ using a resolution of 4 cm^–1^. After purification and digestion of the samples using HNO_3_, the total silver content in the ConA/Ag/AgCl-NPs and *Sa*/Ag/AgCl-NPs were determined by inductively coupled plasma mass spectrometer (ICP–MS-7900, Agilent/Japan). Calibration was performed using a Ag^+^ standard solution from Merck, Germany. Transmission electron microscopy (TEM) was employed for the morphological characterization of the ConA/Ag/AgCl-NPs. The NPs were visualized using a JEOL JEM-1011 instrument, which operated at an accelerating voltage of 60 kV. The gentamicin structure was generated using the ChemDraw program (Revvity Signals Software) and the crystal structure of ConA was generated using the PyMol program (https://www.pymol.org/).

### 2.4 Antibacterial Synergy Assay

For all antibacterial assays shown in this study, a stock solution of ConA/Ag/AgCl-NPs at 448.5 mg/mL (total silver concentration) was prepared in ultrapure water. The concentration of gentamicin (Sigma-Aldrich) was determined using a precision balance and its molar mass. The Gram-positive bacterium *S. aureus* (ATCC 29213) and the Gram-negative *P. aeruginosa* (INCQS 313) were obtained from the Reference Microorganism Collection in Sanitary Surveillance of the Oswaldo Cruz Foundation (FIOCRUZ), Rio de Janeiro, Brazil. The antibacterial activities of gentamicin and ConA/Ag/AgCl-NPs were determined by a microdilution assay.^25^ Peptone broth (PB, 0.5% NaCl, 1% peptone pH 7.4) was used for the antibacterial assays. Microorganism suspensions (1 × 10^3^ CFU/mL) in the mid log growth phase were incubated with 2-fold serial dilutions of gentamicin or ConA/Ag/AgCl-NPs in 96-well microplates at 30 °C for 18 h under gentle stirring. Bacterial cell growth was assessed by measuring the absorbance at 595 nm in a Multiskan Go microplate reader (Thermo Scientific, USA). The minimum inhibitory concentration (MIC) of ConA, gentamicin, and ConA/Ag/AgCl-NPs was defined as the lowest concentration that inhibits at least 90% of the bacterial growth. The positive control (untreated bacteria) was used as a reference for 100% bacterial cell growth.

The synergistic antibacterial effect of the combination of antibiotic gentamicin and ConA/Ag/AgCl-NPs was assessed by the checkerboard titration method.^19,26^ Briefly, stock solutions of ConA and gentamicin were prepared at concentrations of 20 and 5 mg/mL, respectively. For combinations of ConA and gentamicin, final concentrations ranging from 1000 μg/mL to 0.9 μg/mL of ConA and 1.56 μg/mL to 0.2 μg/mL of gentamicin were analyzed. For the synergy assays between gentamicin and ConA/Ag/AgCl-NPs, the final concentrations tested varied between 1.56 μg/mL and 0.2 μg/mL for gentamicin and between 0.35 μg/mL and 0.0003 μg/mL for ConA/Ag/AgCl-NPs.

Data analysis of the checkerboard assay involved calculating the fractional inhibitory concentration (FIC) index, a key metric used to quantitatively assess synergy between the tested antimicrobial agents.^19,26^ The FIC index is calculated by comparing the MIC of each antibacterial compound alone with the MIC obtained when the agents are used in combination. Antibacterial associations were compared with the MIC indexes of the individual antibacterial compounds. The FIC index is used to interpret the interaction between the compounds.^19,26^ FIC indexes are calculated and categorized as follows: FIC index ≤0.5—synergistic; 0.5 < FIC index ≤1.0—nonsynergistic or additive; 1.0 < FIC index ≤4.0—indifferent; FIC index >4.0—antagonistic. All measurements were performed in triplicate, and the averaged values are reported. Based on these criteria, synergistic interactions occur when the combined effect exceeds the sum of individual activities, resulting in a marked reduction in MIC values. Additive (nonsynergistic) interactions correspond to an effect equal to the sum of the activities of the agents without potentiation. Indifferent interactions indicate no significant change in the MIC of the individual agents, while antagonistic interactions occur when the combination reduces the efficacy, resulting in an increase of the MIC values.

### 2.5 Killing Kinetic Assay

A killing kinetic assay was performed to assess the synergistic bactericidal or bacteriostatic effects of low-concentration gentamicin and ConA/Ag/AgCl-NPs against *P. aeruginosa* and *S. aureus*. Bacterial cultures were incubated in peptone broth (PB) containing 0.02 μg of gentamicin and 0.01 μg and 0.0025 μg of ConA/Ag/AgCl-NPs for *S. aureus* and *P. aeruginosa*, respectively, at 30 °C. Assays containing only gentamicin at its minimum inhibitory concentration were also performed under same conditions. Aliquots were taken at specified time points and plated on LB agar to determine the number of colony-forming units (CFUs). Untreated bacteria served as viability controls. Experiments were conducted in triplicate, and the mean values are reported.

### 2.6 Biofilm Formation and Maintenance Inhibition Assays

Biofilm formation and maintenance inhibition assays using ConA/Ag/AgCl-NPs were performed employing previously described methodology with modifications.^27^ Cultures of *P. aeruginosa* and *S. aureus* were grown in LB medium for 18 h at 37 °C under agitation (200 rpm). The cultures were diluted 1:100 to achieve an approximate concentration of 1 × 10^5^ cells/mL in LB medium supplemented with 1 mg/mL dextrose. To evaluate the effect of ConA/Ag/AgCl-NPs on biofilm formation, bacteria were treated with ConA/Ag/AgCl-NPs in 96-well plates at concentrations ranging from 44 μg/mL to 0.0025 μg/mL for *P. aeruginosa* and from 44 μg/mL to 0.005 μg/mL for *S. aureus*, in sextuplicate for both microorganisms. Wells containing only culture medium were used as negative control. Conversely, untreated bacteria, exhibiting full biofilm formation, were designated as the positive control. The bacteria were incubated at 37 °C for 24 h. Subsequently, the supernatant from each well was collected and plated on LB supplemented with 2% agar to assess bacterial viability. The formed biofilms were washed four times with distilled water and then dried at 60 °C for 2 h. After drying, 100 μL of 0.06% (w/v) crystal violet was added to each well and removed after 10 min. The biofilms were washed four times with distilled water, and 30% (v/v) acetic acid was used to solubilize the dye. The solubilized crystal violet from each well was transferred to a new 96-well plate, and the absorbance was measured at 595 nm using a microplate reader. Increases in absorbance compared to the negative control indicated biofilm formation. Additional plates were prepared in parallel for viable cell counts in biofilms. To achieve this, sterile tips were used to remove the biofilms, and the detached cells were suspended in 0.9% saline solution and vortexed before plating on LB agar.

A biofilm maintenance assay was conducted to evaluate the effect of ConA/Ag/AgCl-NPs on established biofilms. Cultures of *P. aeruginosa* and *S. aureus* were incubated under agitation (200 rpm) at 37 °C for 18 h. Following incubation, cultures were diluted to approximately 1 × 10^5^ cells/mL in LB medium containing 1 mg/mL dextrose, then dispensed into 96-well plates and incubated at 37 °C for 24 h to allow biofilm formation. Subsequently, ConA/Ag/AgCl-NPs treatments were applied in sextuplicate, using a concentration range from 44 μg/mL to 0.0025 μg/mL for *P. aeruginosa* and from 44 μg/mL to 0.005 μg/mL for *S. aureus*. The remaining steps and controls of the experiment were performed as previously described for the biofilm formation assay.

### 2.7 Statistical Analysis

Statistical analysis was performed using GraphPad Prism 5 software. Results were compared using a one-way ANOVA test, and statistical significance was determined by Tukey’s Multiple Comparison Test.

## 3 Results and Discussion

### 3.1 Synthesis and Characterization of ConA-Coated Silver/Silver Chloride NPs (ConA/Ag/AgCl-NPs)

Figure 1A,B depict the structures of gentamicin and ConA, respectively. Gentamicin, an antibiotic that inhibits bacterial protein synthesis, is used to treat infections caused by mycobacteria, enterococci, and various Gram-negative bacteria.^28^ ConA, a tetrameric protein that binds to glucose/mannose saccharides present on the cell surface,^29^ was associated with Ag/AgCl-NPs to form ConA/Ag/AgCl-NPs using a green photochemical approach, as described previously.^21,25^ Figure 1C shows the synthesis process before (vial 1) and after (vial 2) irradiation with visible light, where the color change of the solution from transparent to brown visually shows the formation of ConA/Ag/AgCl-NPs. TEM analyses showed a spherical morphology for the ConA/Ag/AgCl-NPs (inset of Figure 1C), consistent with published results.^21^ The hydrodynamic radius, the mean diameter, and the zeta-potential of ConA/Ag/AgCl-NPs determined by DLS (Figure S1A), TEM (inset of Figure 1C) and ELS (Figure S1B) were 72 ± 2 nm, 44 ± 25 nm, and −24 ± 1 mV, respectively, values in agreement with our previously published results.^21^ The FTIR spectrum of the ConA (Figure S2, black line) shows a band at 1638 cm^–1^ characteristics of the regular β-sheet secondary structure, specifically the amide I (C=O stretching) vibration.^6^ The band at 1526 cm^–1^ is attributed to N–H bending vibrations coupled with C–N stretching vibrations. A broad absorption band centered at 3270 cm^–1^ corresponds to the stretching vibration of O–H bonds in proteins, confirming the presence of intermolecular hydrogen bonds originating from NH_2_ and OH groups in lectins.^6^ The FTIR spectrum of the ConA/Ag/AgCl-NPs (Figure S2, red line) showed no significant differences when compared to the isolated ConA, exhibiting approximately the same band pattern. Therefore, these results clearly demonstrated the association of ConA with Ag/AgCl-NPs.

**Figure 1:**
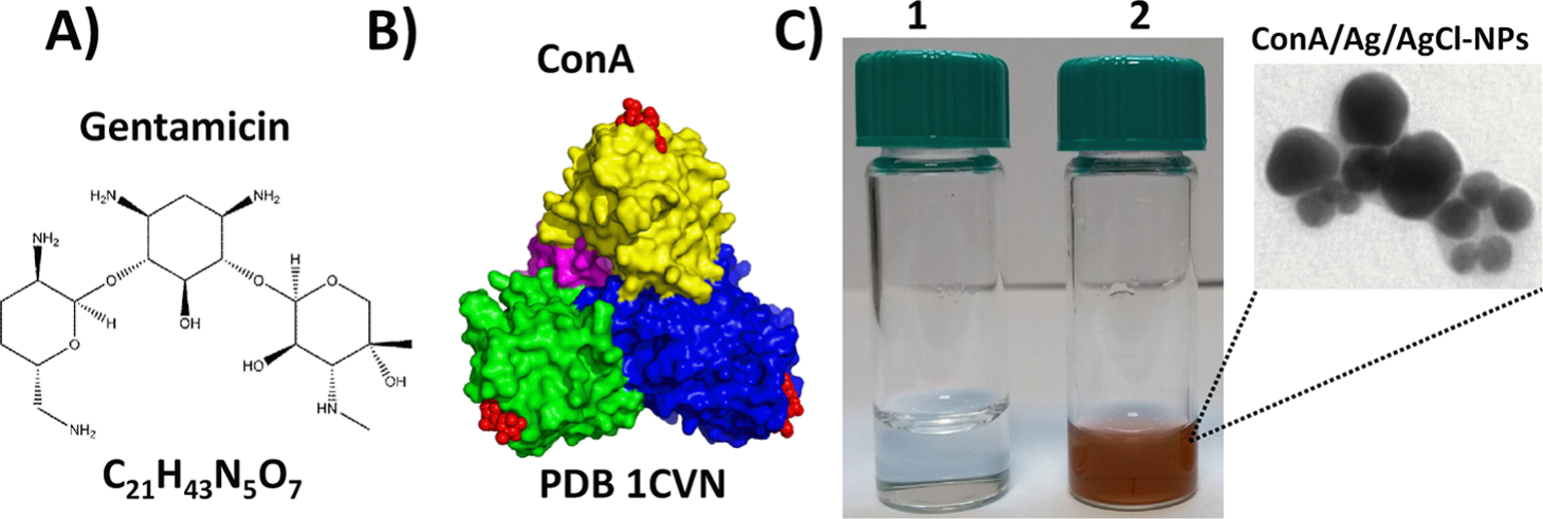
(A) Molecular structure of gentamicin (477.56 g/mol). (B) Tetrameric crystallographic structure of ConA (PDB ID: 1CVN) with the trimannoside molecules bound to the carbohydrate recognition domain (CRD) highlighted in red. (C) Light-mediated synthesis of ConA/Ag/AgCl-NPs. Vial 1 (control): 1 mM Tris-HCl buffer at pH 8 containing 1 mM AgNO_3_. Vial 2:1 mM Tris-HCl buffer at pH 8 containing 1 mM AgNO_3_ and 0.1 mg/mL of ConA. Inset: transmission electron micrograph (TEM) showing purified ConA/Ag/AgCl-NPs with the thin layer of ConA coating the surface of the NPs.

### 3.2 Analysis of the Interaction Between Gentamicin and ConA/Ag/AgCl-NPs

A recent study demonstrated that gentamicin binds to the carbohydrate recognition domain (CRD) of ConA.^5^ Furthermore, it was reported that ConA/Ag/AgCl-NPs retain their ability to agglutinate rabbit erythrocytes, indicating that ConA’s CRD remains functional on the NPs surface, allowing it to interact with biological membrane carbohydrates and potentially even with antibiotics.^21^

To investigate the interaction between gentamicin and ConA/Ag/AgCl-NPs, we monitored the surface plasmon resonance (SPR) spectrum of the ConA/Ag/AgCl-NPs as a function of increasing gentamicin concentration (Figure 2). The SPR spectrum is highly sensitive to modifications on the surface of NPs.^30,31^ No significant absorption was observed for gentamicin, even at the highest concentration utilized. The absorption spectrum of ConA/Ag/AgCl-NPs exhibited a strong SPR band between 400 and 600 nm (Figure 2A), characteristic of AgNPs, consistent with our previously published results.^21^ Increasing gentamicin concentration resulted in a progressive decrease in the intensity of the absorption spectrum as well as a red-shift. The observed linear decrease in intensity and red-shift of the SPR spectrum are characteristic indicators of molecular binding events on the surface of the ConA/Ag/AgCl-NPs.^30,31^ Specifically, the red-shift is primarily attributed to an increase in the local refractive index caused by the added mass of the bound substrate.^30,31^ The decrease in intensity, on the other hand, can be attributed to increased damping of plasmon oscillations and potential conformational changes within the protein layer upon substrate binding.

**Figure 2:**
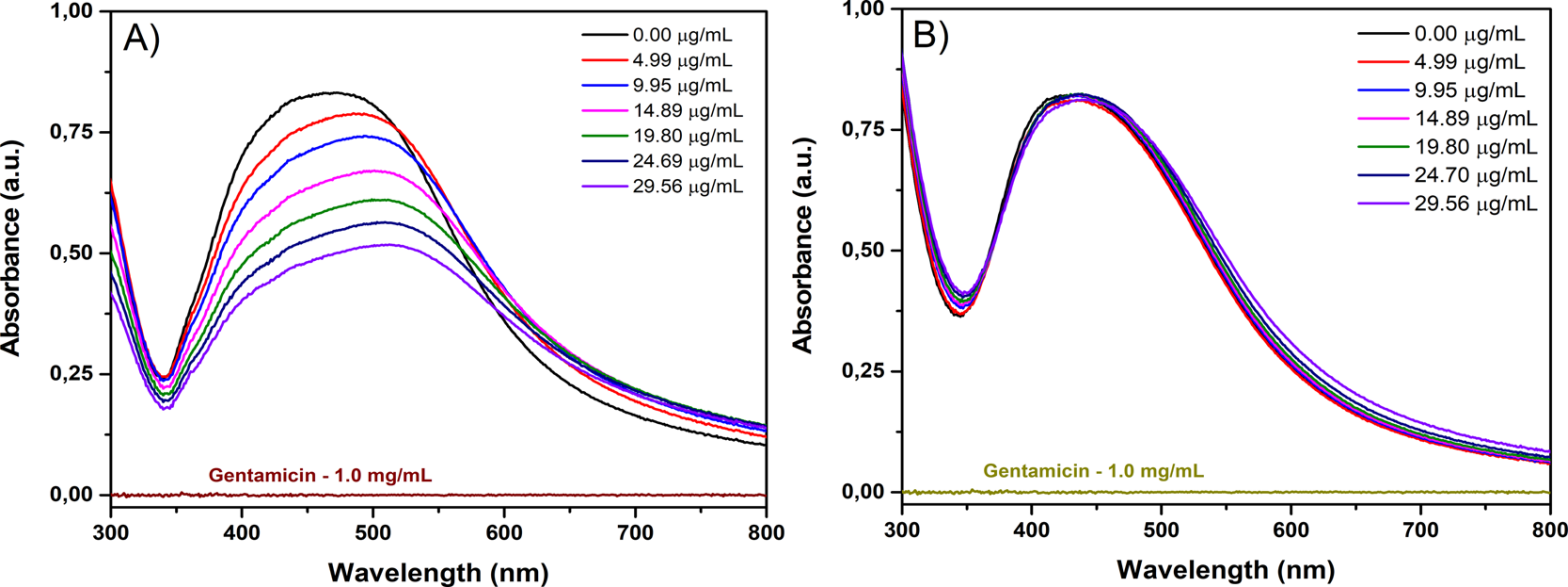
Absorption spectra of functionalized Ag/AgCl-NPs in the presence of gentamicin. (A) Absorption spectrum of ConA/Ag/AgCl-NPs in the absence and presence of increasing concentrations of gentamicin. (B) Absorption spectrum of *Sa*/Ag/AgCl-NPs in the absence and presence of increasing concentrations of gentamicin.

In contrast to the ConA/Ag/AgCl-NPs, increasing gentamicin concentration did not induce any significant change in the SPR spectrum of the Ag/AgCl-NPs coated with a nonantimicrobial plant extract from *S. adstringens* (*Sa*/Ag/AgCl-NPs), indicating a lack of interaction (Figure 2B).^25^ ConA/Ag/AgCl-NPs and *Sa*/Ag/AgCl-NPs exhibit similar physicochemical properties, including size, shape, net charge, and inorganic chemical composition, with the only difference being their organic stabilizing component.^21,25^

### 3.3 Minimum Inhibitory Concentrations of ConA, Gentamicin, and ConA/Ag/AgCl-NPs

The antibacterial activities of ConA, gentamicin, and ConA/Ag/AgCl-NPs were evaluated by a microdilution assay against the pathogenic bacteria *S. aureus* (Gram-positive) and *P. aeruginosa* (Gram-negative). Even at 1000 μg/mL, the highest concentration tested, ConA was not able to inhibit bacterial growth, indicating an absence of antibacterial activity when tested alone against the two bacterial strains (Figure S3). Our findings are consistent with a recent study, which reported that ConA lacked inhibitory activity against multidrug-resistant (MDR) bacteria even at a final concentration of 1024 μg/mL.^5^ Conversely, as expected, gentamicin displayed high antibacterial activity against *S. aureus* and *P. aeruginosa* with MIC values of 0.19 μg/mL and 0.39 μg/mL, respectively (Figures S4–S6 and Table 1).

**Table 1:** Growth Inhibitory Effects of the Gentamicin and ConA/Ag/AgCl-NPs Alone or in Combination against *S. aureus* and *P. aeruginosa* Strains, Respectively

*Staphylococcus aureus*			
antibacterial agent	MIC^a^ (μg/mL) (alone)	MIC^a^ (μg/mL) (combination)	FIC^b^ (combination/alone)
gentamicin	0.19	0.02	0.10
ConA/Ag/AgCl-NPs	0.04	0.01	0.25
final FIC = 0.10 + 0.25 = 0.35			

*Pseudomonas aeruginosa*			
antibacterial agent	MIC^a^ (μg/mL) (alone)	MIC^a^ (μg/mL) (combination)	FIC^b^ (combination/alone)
gentamicin	0.39	0.02	0.05
ConA/Ag/AgCl-NPs	0.02	0.0012	0.06
final FIC = 0.05 + 0.06 = 0.11			

a MIC—minimal inhibitory concentration that inhibits at least 90% of the bacterial growth.

b FIC—fractional inhibitory concentration. FIC indexes ≤0.5 indicates that the two compounds work synergistically.

For all antibacterial assays using ConA/Ag/AgCl-NPs, a stock solution with a total silver concentration of 448.5 mg/mL was prepared in ultrapure water. This stock solution was the basis for the dilutions used in the subsequent experiments. ConA/Ag/AgCl-NPs demonstrated remarkable growth inhibitory activity against *S. aureus* and *P. aeruginosa*, with MIC values of 0.04 μg/mL and 0.02 μg/mL, respectively (Figures 3, S7, S8 and Table 1).

**Figure 3:**
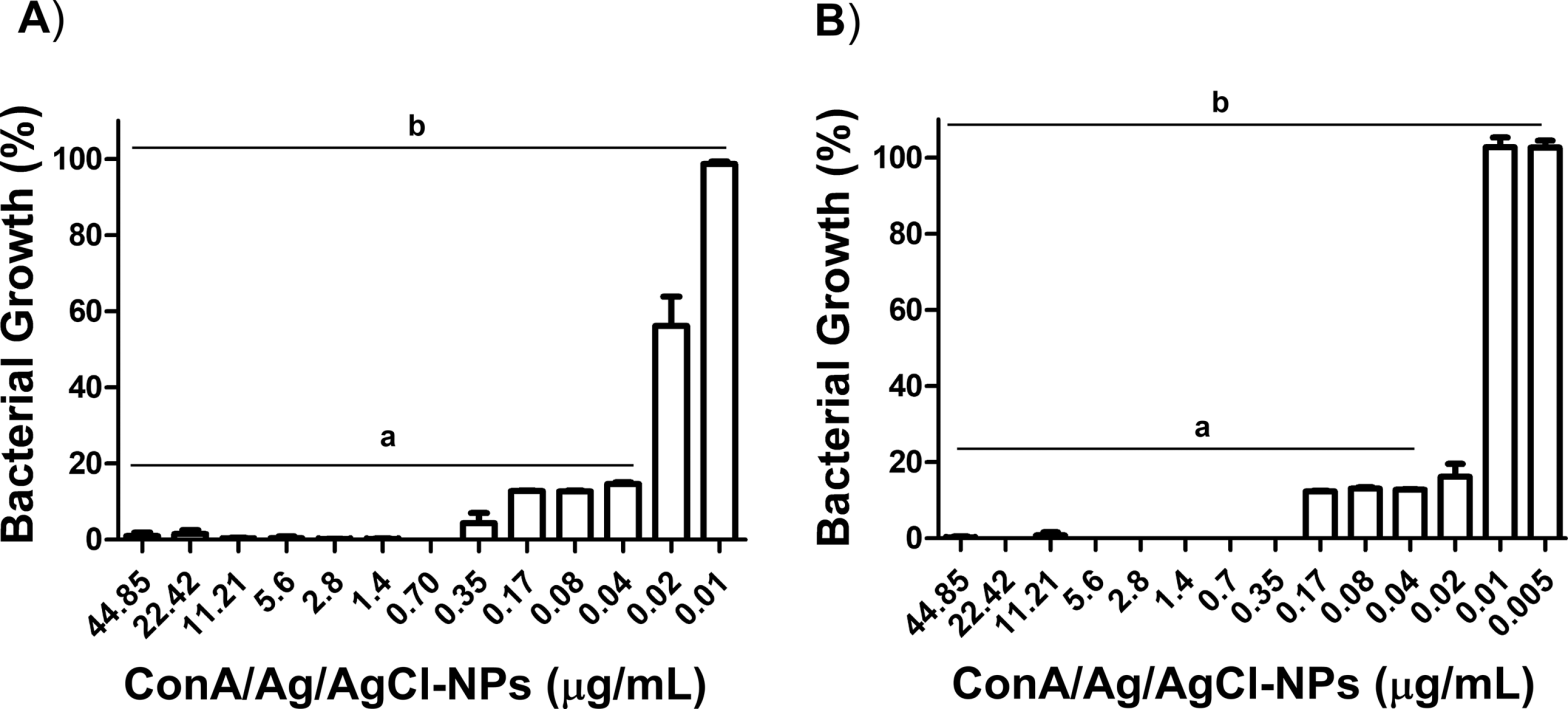
Antibacterial activity of ConA/Ag/AgCl-NPs against *S. aureus* (A) and *P. aeruginosa* (B). Results were compared by the one-way ANOVA test and the statistical significance was determined by Tukey’s test, with *p* < 0.05. The groups represented in “a” did not show statistically significant differences between them, and the groups represented in “b” show statistically significant differences.

### 3.4 Synergistic Effect of the Gentamicin and ConA/Ag/AgCl-NPs Combination

The combined antibacterial activity of gentamicin with ConA or ConA/Ag/AgCl-NPs was evaluated against the pathogenic bacteria *S. aureus* and *P. aeruginosa* at noninhibitory concentrations. While the use of lectins as adjuvants for synergistic antibiotic therapies has been explored, further research in this area is highly warranted.^32^ No significant change in the MIC value of gentamicin was observed against *P. aeruginosa* when combined with ConA, even at 1000 μg/mL, which is consistent with our previously reported results (Figure S9).^5^ However, ConA at 1000 μg/mL decreased the MIC of gentamicin against *S. aureus* from 0.19 μg/mL to 0.08 μg/mL (Figure S10), a significant 58% reduction in the amount of gentamicin needed to achieve the same inhibitory effect against *S. aureus*. These findings support previously published results demonstrating enhanced gentamicin activity against *S. aureus* when combined with ConA.^5^ In this previous study, when combined with gentamicin, ConA reduced the MIC value of gentamicin from 64.0 μg/mL to 12.7 μg/mL against MDR *S. aureus* (about 5-fold).^5^ This finding indicates an interaction between ConA and gentamicin, which is possible through the CRD, as also evidenced by previous hemagglutination inhibition assays.^21^

The impact of lectins on antibiotic efficacy varies depending on the specific lectin and bacterial strain. For instance, *Dioclea violacea* lectin (DVL) significantly reduces the MIC of gentamicin against *S. aureus* from 50.8 to 10.1 μg/mL (about 5-fold).^22^ In addition, *Parkia platycephala* lectin (PPL) reduces the MIC of gentamicin against *S. aureus*, resulting in a 61% decrease.^33^ Interestingly, PPL does not enhance gentamicin activity against *P. aeruginosa*, a finding also observed for ConA and DVL. This limited effect might be due to the unique extracellular polysaccharides produced by *P. aeruginosa*, which could potentially hinder gentamicin penetration. However, further investigations are warranted to elucidate the precise mechanisms underlying this interaction.^32^

ConA itself lacks direct antibacterial activity, but its high specificity for glucose and mannose sugars on bacterial surfaces makes it a valuable tool for targeted delivery in combination therapies. Against *S. aureus*, combining gentamicin with ConA/Ag/AgCl-NPs reduced the required concentrations from 0.19 μg/mL and 0.04 μg/mL to 0.02 μg/mL and 0.01 μg/mL, respectively (Table 1 and Figure 4). This translates to reductions of 89.5% and 75% in the amount of gentamicin and ConA/Ag/AgCl-NPs needed, respectively. The FIC index of 0.35 (Table 1) indicates synergistic antibacterial activity between gentamicin and ConA/Ag/AgCl-NPs. An FIC value of 0.5 or less is widely accepted as the threshold for synergy.^19,26^ This finding contrasts with the study of Abo-Shama et al.,^34^ where no synergistic effect was observed between gentamicin and AgNPs biosynthesized using *Ulva fasciata* aqueous extract.

**Figure 4:**
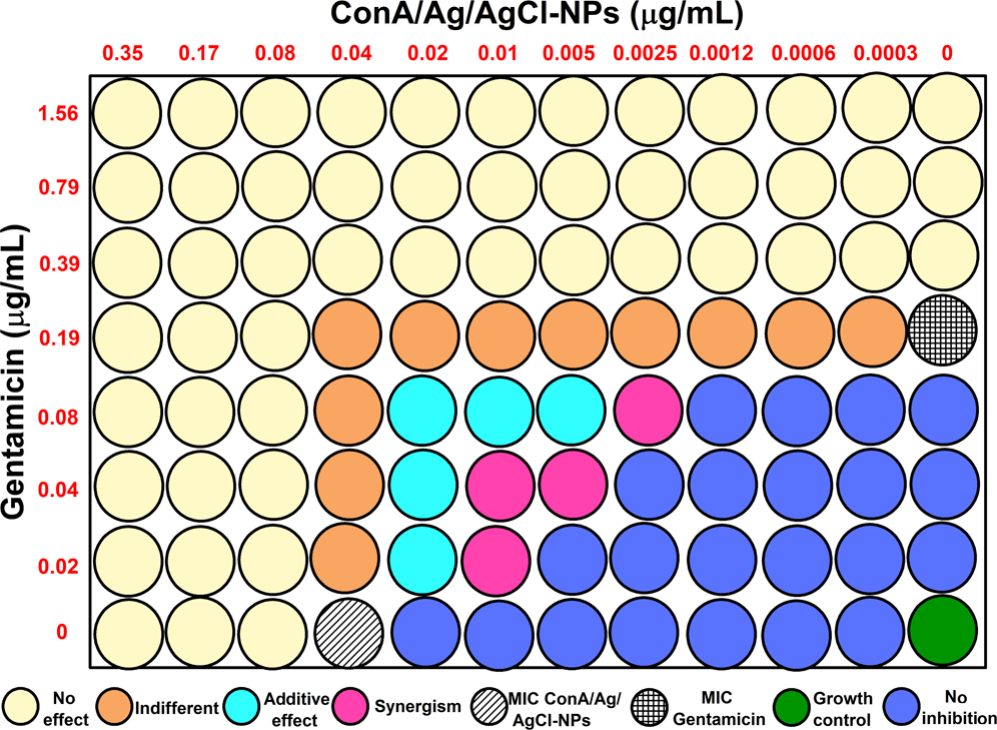
Illustration of the 96-well plate for the synergism between gentamicin and ConA/Ag/AgCl-NPs for *S. aureus*. The yellow circles correspond to concentrations that had no effect with FIC index >2.0. The orange circles correspond to concentrations that had an indifferent effect (1.0 < FIC index < 2.0), the cyan circles correspond to concentrations that had an additive effect (0.5 < FIC index < 1.0), and pink circles correspond to concentrations that demonstrated a synergistic effect (FIC index < 0.5).

Furthermore, the synergistic effect between AgNPs and the antibiotics gentamicin and neomycin were investigated against 20 *S. aureus* strains, bacteria involved in the development of mastitis, isolated from cow’s milk.^35^ They found that 55% of the strains were resistant to gentamicin, but combining it with AgNPs reduced the percentage of resistant strains by 15%. Notably, a synergistic effect between gentamicin and AgNPs was observed in half (10 out of 20) of the isolated strains, indicating that this combination is a potential treatment for *S. aureus*-induced mastitis.

Combining gentamicin with ConA/Ag/AgCl-NPs against *P. aeruginosa* reduced the required concentrations from 0.39 μg/mL and 0.02 μg/mL to 0.02 μg/mL and 0.0012 μg/mL, respectively (Table 1 and Figure 5). This combination achieved a remarkable 94.9% reduction in gentamicin and a 94.0% reduction in ConA/Ag/AgCl-NPs required to inhibit *P. aeruginosa*, in contrast to the results described for PPL lectin.^33^ The FIC index of 0.11 (Table 1) indicates synergistic antibacterial activity between gentamicin and ConA/Ag/AgCl-NPs.

**Figure 5:**
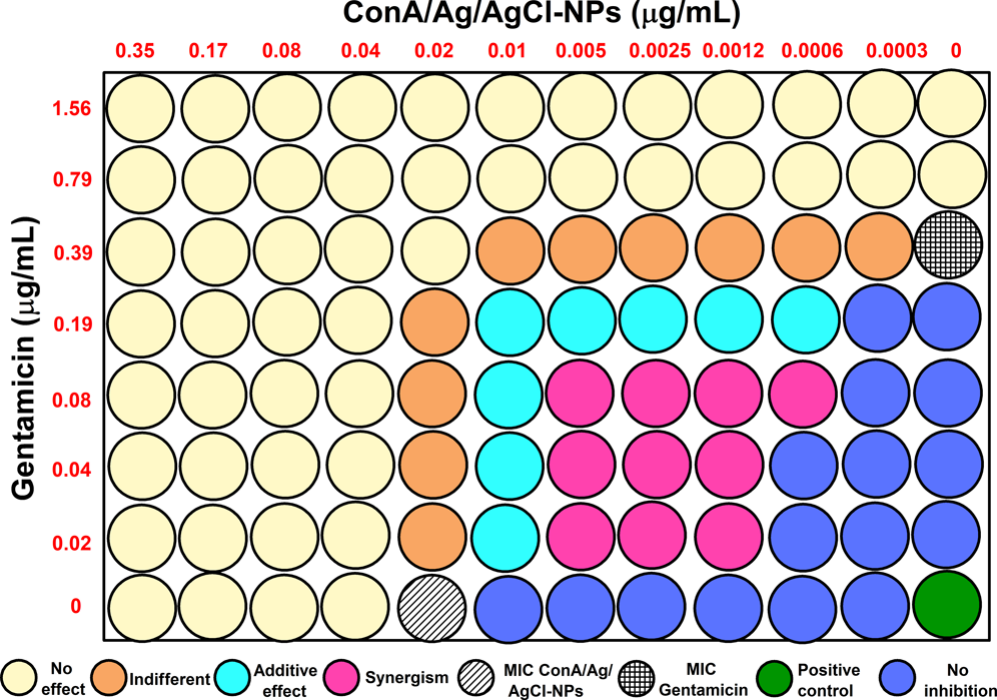
Illustration of the 96-well plate for the synergism between gentamicin and ConA/Ag/AgCl-NPs for *P. aeruginosa*. The yellow circles correspond to concentrations that had no effect with FIC index > 2.0. The orange circles correspond to concentrations that had an indifferent effect (1.0 < FIC index < 2.0), the cyan circles correspond to concentrations that had an additive effect (0.5 < FIC index < 1.0), and pink circles correspond to concentrations that demonstrated a synergistic effect (FIC index < 0.5).

Previously reported TEM analysis revealed a higher probability of adhesion of AgNPs stabilized with poly­(*N*-vinyl-2-pyrrolidone) (PVP) when combined with gentamicin, compared to NPs alone. This result suggests that gentamicin enhances the attachment of PVP/AgNPs to the bacterial surface, likely leading to increased antibacterial activity.^36^ Additionally, the authors speculate that aggregates of PVP/AgNPs on the bacterial surface might facilitate gentamicin internalization through adsorption. However, further studies are needed to elucidate the specific behavior of gentamicin in these circumstances.

Consistent with the synergistic activity of ConA/Ag/AgCl-NPs with gentamicin observed in this work, and other studies demonstrating the effect of plant lectins on gentamicin action and their interaction via CRD,^33^ additional studies report that plant lectins can enhance the activity of other antibiotics besides aminoglycosides, including ceftazidime,^37^ norfloxacin, and penicillin.^38^ These results suggest that lectins and their functionalization with NPs can be promising adjuvants to enhance antibiotic action against microbial infections.

Silver halide NPs are thermodynamically unstable and prone to aggregation without a stabilizing agent. Although sodium citrate is commonly used to stabilize “bare” silver-based NPs, citrate-capped silver-based NPs are unsuitable controls for our study due to their inherent cytotoxicity.^39,40^ Consequently, we used *Sa*/Ag/AgCl-NPs as a control, synthesized^25^ using the same methodology previously described for ConA/Ag/AgCl-NPs.^25^ We evaluated the combined antibacterial activity of gentamicin with *Sa*/Ag/AgCl-NPs against *S. aureus* and *P. aeruginosa*. Notably, no synergistic effect was observed in the combination of *Sa*/Ag/AgCl-NPs with gentamicin. The calculated FIC indexes were greater than 1.0 in both cases, indicating an indifferent effect, or absence of a synergistic effect (Figures S11 and S12). The absence of synergy likely stems from the lack of interaction between gentamicin and *Sa*/Ag/AgCl-NPs, as demonstrated earlier (Figure 2). Comparable behavior was observed in the combination of gentamicin and AgNPs biosynthesized using an *U. fasciata* aqueous extract.^34^ Therefore, these results underscore the essential role of ConA lectin in the synergistic effect observed with the combination of gentamicin and ConA/Ag/AgCl-NPs.

### 3.5 Bactericidal Effect of the Gentamicin and ConA/Ag/AgCl-NPs Combination

The bactericidal activity of gentamicin and ConA/Ag/AgCl-NPs against *P. aeruginosa* and *S. aureus* was evaluated. Gentamicin and ConA/Ag/AgCl-NPs demonstrated potent synergistic bactericidal activity against *S. aureus* (Figure 6A) and *P. aeruginosa* (Figure 6B), even at sub-MIC concentrations.

**Figure 6:**
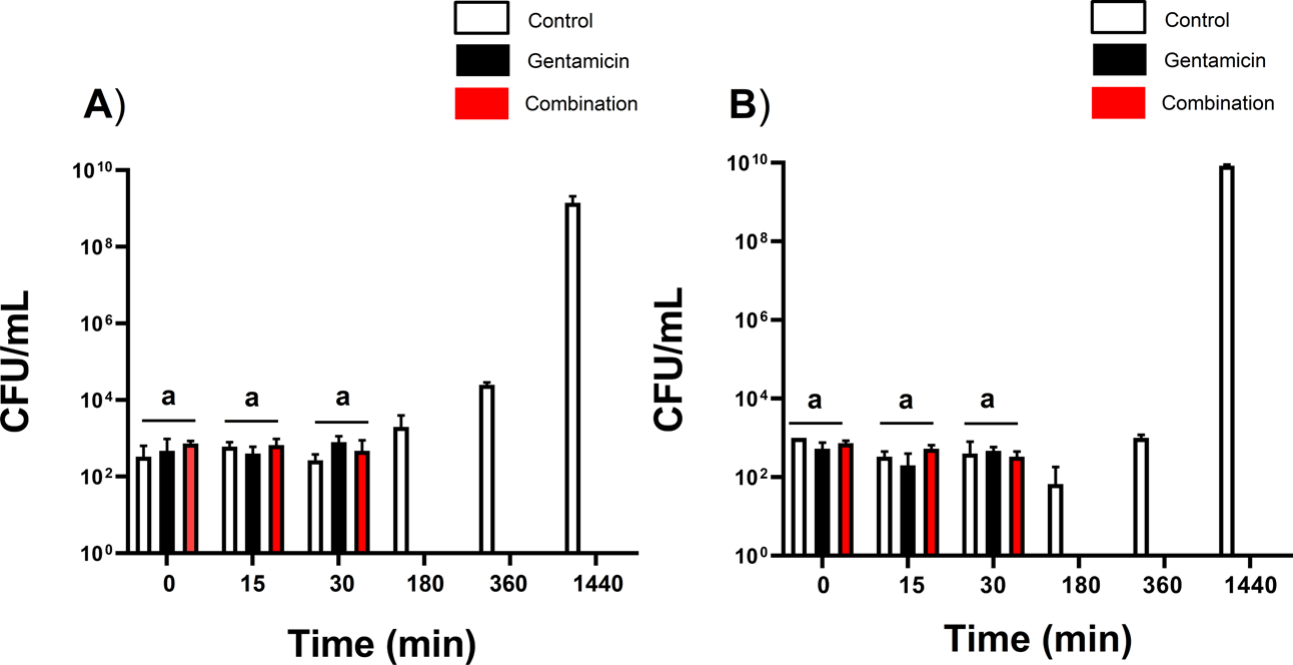
Killing kinetic assays for *S. aureus* (A) and *P. aeruginosa* (B) in the presence of gentamicin and ConA/Ag/AgCl-NPs at synergistic concentrations. The white bars represent the control group, while black bars indicate the assay performed with gentamicin at its minimum inhibitory concentration (MIC of 0.39 μg/mL and 0.19 μg/mL of gentamicin for *P. aeruginosa* and *S. aureus*, respectively). Red bars correspond to the combined treatment with gentamicin (0.02 μg/mL) and ConA/Ag/AgCl-NPs (0.0025 μg/mL and 0.01 μg/mL for *P. aeruginosa* and *S. aureus*, respectively). The groups represented in “a” did not show statistically significant differences between them.

This was evidenced by a significant reduction in bacterial cell count within 180 min for both bacteria compared to the control group. Despite identical exposure times for complete bacterial death in both Gram-positive and Gram-negative bacteria (180 min for both), differences in cellular composition may still influence the interaction between gentamicin and ConA/Ag/AgCl-NPs. Importantly, this study demonstrates that combining gentamicin with ConA/Ag/AgCl-NPs significantly reduces the required concentrations to achieve the same effective antibacterial activity against both *S. aureus* and *P. aeruginosa*. Previous studies have suggested that the bactericidal effects of AgNPs on different strains may involve mechanisms such as reactive oxygen species (ROS) induction, disruption of bacterial enzymes, damage to biomolecules, and targeting of the bacterial membrane.^41–43^ However, further research is necessary to fully understand the specific mechanisms underlying this synergistic interaction.

### 3.6 Effect of Inhibition of Biofilm Formation by ConA/Ag/AgCl-NPs

A biofilm is a surface-attached community of bacterial cells, encased in a protective, hydrated matrix of extracellular polymeric substances. Critically, these biofilm-embedded bacteria demonstrate a profound resistance to antimicrobial agents, with tolerances 10 to 1000-fold higher than free-floating (planktonic) cells.^44,45^ To gain further insight into the mechanism of action of ConA/Ag/AgCl-NPs, assays were performed to evaluate their impact on biofilms naturally produced by *S. aureus* and *P. aeruginosa*. These biofilm formation inhibition assays employed a wide range of concentrations, encompassing values both above and below the established MICs. Our results showed that *S. aureus* displayed substantial reduction in biofilm formation at 44 μg/mL and 22 μg/mL (value at least 500-fold greater than that observed for planktonic cells, Table 1), with only about 10% remaining compared to the control. At 11 μg/mL, biofilm formation increased to roughly 20%. Notably, at lower concentrations, ranging from 1.35 μg/mL to 0.0025 μg/mL, biofilm formation approached 100% of the control (Figure 7A and Figure S13A).

**Figure 7:**
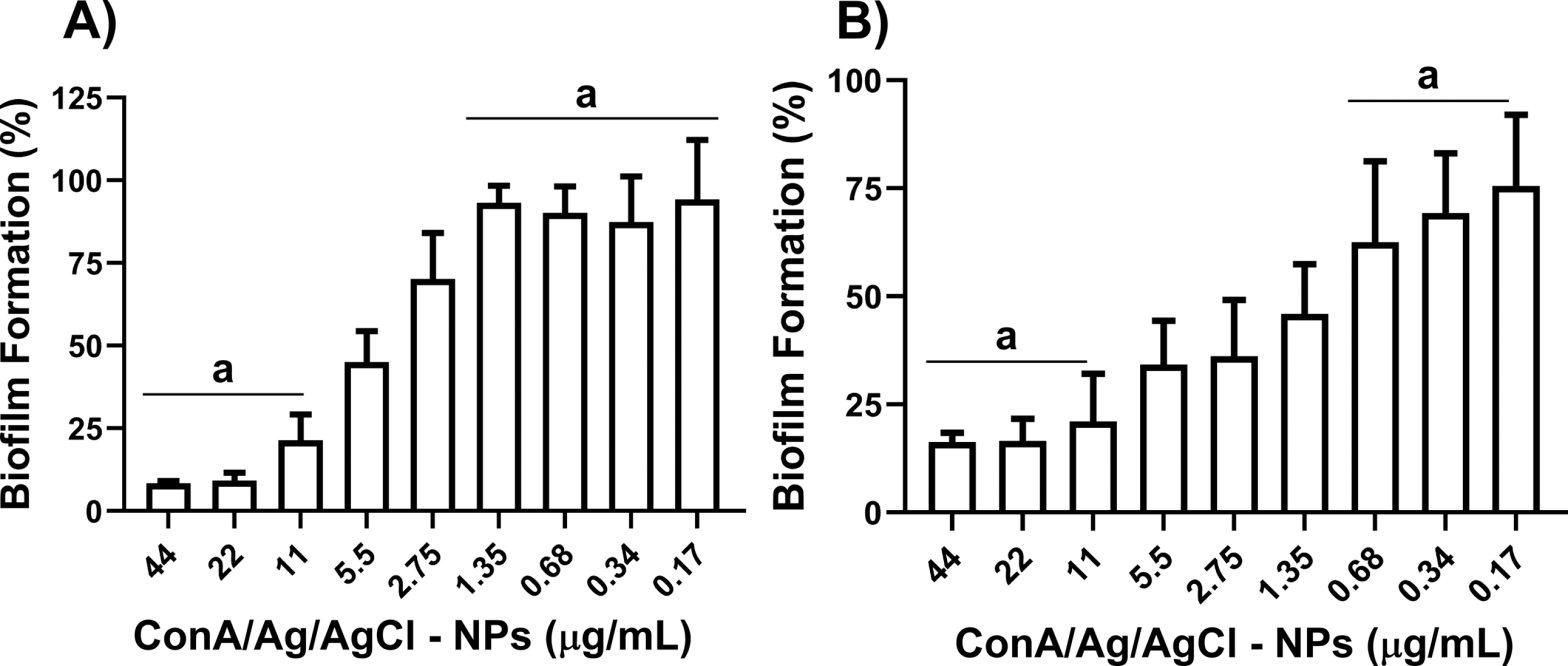
Effect of ConA/Ag/AgCl-NPs on biofilm formation against *S. aureus* (A) and *P. aeruginosa* (B). Results were compared by the one-way ANOVA test and the statistical significance was determined by Tukey’s test, with *p* < 0.05. The groups represented in “a” did not show statistically significant differences between them.

Conversely, *P. aeruginosa* demonstrated distinct biofilm formation patterns. Concentrations of 44 μg/mL and 22 μg/mL resulted in the biofilm formation of approximately 25% when compared to the control. Within the concentration range of 5.5 μg/mL to 1.35 μg/mL, biofilm formation reached approximately 50%. Higher levels of biofilm formation were only observed at concentrations below 0.68 μg/mL (Figures 7B and S13B).

In a parallel assay, the capacity of ConA/Ag/AgCl-NPs to degrade established biofilms was evaluated. Sensitivity of *P. aeruginosa* was observed solely at 44 μg/mL, leading to 50% biofilm formation relative to the control (Figure 8B). Conversely, *S. aureus* exhibited nearly 100% biofilm formation at this same concentration (Figure 8A).

**Figure 8:**
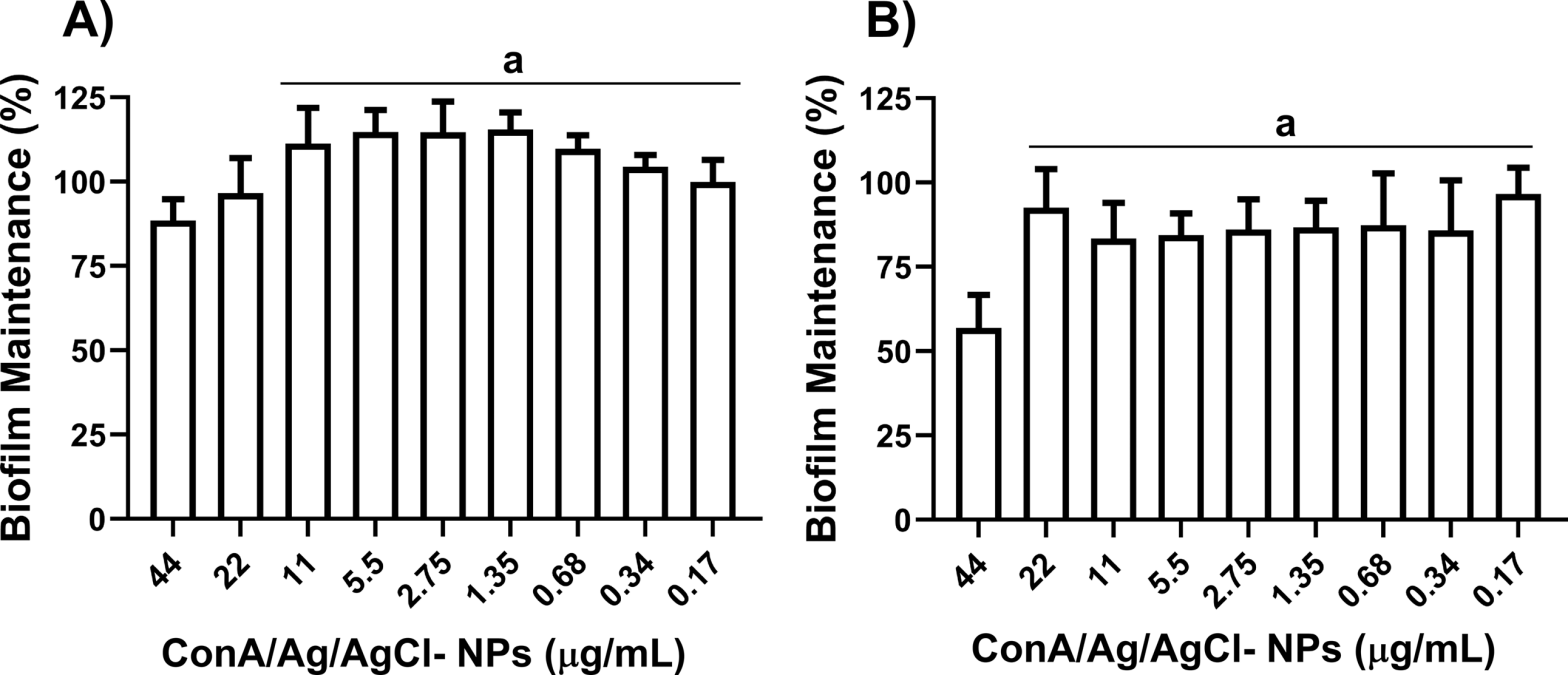
Effect of ConA/Ag/AgCl-NPs on biofilm maintenance against *S. aureus* (A) and *P. aeruginosa* (B). Results were compared by the one-way ANOVA test and the statistical significance was determined by Tukey’s test, with *p* < 0.05. The groups represented in “a” did not show statistically significant differences between them.

At all subsequent lower concentrations tested, both bacterial strains displayed 100% biofilm formation, indicating that ConA/Ag/AgCl-NPs did not exert substantial degradative effect under these conditions (Figures 8 and S14). These findings suggest a potential direct interaction between ConA/Ag/AgCl-NPs and the bacterial cell wall. Furthermore, it is plausible that higher concentrations of ConA/Ag/AgCl-NPs release greater amounts of Ag^+^ ions, leading to significant inhibition of biofilm formation.^46^ Indeed, we observed that most effective concentrations of ConA/Ag/AgCl-NPs also reduced the number of viable cells in both bacterial strains (Figures S15 and S16), suggesting that damaged or dead cells have a reduced ability to aggregate and form a structured biofilm. Nevertheless, additional mechanistic investigations are necessary to derive more complete conclusions.

## 4 Conclusion

In summary, our results confirmed that gentamicin interacts with ConA/Ag/AgCl-NPs, thereby altering their plasmonic resonance properties. Furthermore, our findings demonstrate a significant synergistic effect and bactericidal activity between gentamicin and ConA/Ag/AgCl-NPs against the pathogenic planktonic bacteria *S. aureus* and *P. aeruginosa*. The MIC of gentamicin required for inhibition of bacterial growth drastically decreased when combined with ConA/Ag/AgCl-NPs, resulting in a reduction in the amount of antibiotic needed. This translates to a reduction of over 90% in gentamicin usage for both bacterial strains. Furthermore, FIC indexes confirmed the synergistic nature of these combinations. This implies that ConA/Ag/AgCl-NPs can potentiate the action of gentamicin, allowing for effective bacterial control at lower antibiotic doses in planktonic conditions. In contrast, substantial biofilm reduction by ConA/Ag/AgCl-NPs required concentrations at least 500-fold greater than the MICs observed for inhibition of planktonic cells. Therefore, these results hold significant promise for the development of combination therapies utilizing ConA/Ag/AgCl-NPs alongside gentamicin against pathogenic planktonic bacteria. For example, incorporating gentamicin and ConA/Ag/AgCl-NPs into a film or ointment could offer a promising antibacterial approach for external applications. By reducing the reliance on high antibiotic doses, such strategies could potentially mitigate the emergence of antibiotic-resistant bacteria. Further investigations are warranted to evaluate the efficacy and safety of this combination therapy, paving the way for a potential weapon in the fight against antibiotic resistance.

## Supplementary Material



## Notes

Characterization of ConA/Ag/AgCl-NPs using DLS and ELS (Figure S1); Fourier transform infrared spectroscopy analysis of ConA and ConA/Ag/AgCl-NPs (Figure S2); antibacterial activity of ConA against *S. aureus* and *P. aeruginosa* (Figure S3); antibacterial activity of gentamicin against *S. aureus* and *P. aeruginosa* (Figure S4); illustration of a 96-well plate used to determine the minimum inhibitory concentration (MIC) of gentamicin against *S. aureus* (Figure S5); illustration of a 96-well plate used to determine the minimum inhibitory concentration (MIC) of gentamicin against *P. aeruginosa* (Figure S6); illustration of a 96-well plate used to determine the minimum inhibitory concentration (MIC) of ConA/Ag/AgCl-NPs against *S. aureus* (Figure S7); illustration of a 96-well plate used to determine the minimum inhibitory concentration (MIC) of ConA/Ag/AgCl-NPs against *P. aeruginosa* (Figure S8); illustration of a 96-well plate used to assess the synergistic interaction between gentamicin and ConA protein against *P. aeruginosa* (Figure S9); illustration of a 96-well plate used to assess the synergistic interaction between gentamicin and ConA protein against *S. aureus* (Figure S10); illustration of a 96-well plate used to assess the synergistic interaction between gentamicin and Sa/Ag/AgCl-NPs protein against *S. aureus* (Figure S11); illustration of a 96-well plate used to assess the synergistic interaction between gentamicin and *Sa*/Ag/AgCl-NPs protein against *P. aeruginosa* (Figure S12); effect of ConA/Ag/AgCl-NPs on biofilm formation against *S. aureus* and *P. aeruginosa* at lower concentrations (Figure S13); effect of ConA/Ag/AgCl-NPs on biofilm maintenance against *S. aureus* and *P. aeruginosa* at lower concentrations (Figure S14); the number of viable *S. aureus* cells (CFU/mL) in biofilms and planktonic cell suspensions after treatment with ConA/Ag/AgCl-NPs (Figure S15) and the number of viable *P. aeruginosa* cells (CFU/mL) in biofilms and planktonic cell suspensions after treatment with ConA/Ag/AgCl-NPs (PDF)
