# Supplementary material for: Lectin-Coated Silver/Silver Chloride Nanoparticles in Combination with Gentamicin: A Strategy to Preserve Antibiotic Efficacy at Lower Doses Against Pathogenic Planktonic Bacteria
Source: ACS Omega. 2025 Nov 24;10(48):58164–74. doi: 10.1021/acsomega.5c03081 (PMC12771260; doi:10.1021/acsomega.5c03081)

Supplementary Material

Lectin-coated silver/silver chloride nanoparticles in combination with gentamicin: a strategy to preserve antibiotic efficacy at lower doses against pathogenic planktonic bacteria

Viviane Brito Andrade[a], Diógenes G. da S. Fernandes[a], Dnane Vieira Almeida[a], Geomar F. Cruz[a], Tamara Jarosi Handajevsky[b], Daiany A. Ribeiro[c], Claudener S. Teixeira[c], André Luis Coelho da Silva[d], Fernanda Dias da Silva[b], Wanius Garcia[a,*]

[a] Centro de Ciências Naturais e Humanas, Universidade Federal do ABC (UFABC), Santo André, SP, Brazil.

[b] Centro de Ciências Naturais e Humanas, Universidade Federal do ABC (UFABC), São Bernardo do Campo, SP, Brazil.

[c] Centro de Ciências Agrárias e da Biodiversidade, Universidade Federal do Cariri (UFCA), Crato, CE, Brazil.

[d] Laboratório de Biotecnologia Molecular (LabBMol), Departamento de Bioquímica e Biologia Molecular, Universidade Federal do Ceará, Fortaleza, CE, Brazil.

[*] Corresponding author: wanius.garcia@ufabc.edu.br (W. Garcia).

Table of contents

Figure S1. Characterization of ConA/Ag/AgCl-NPs using DLS and ELS. **03**

Figure S2. Fourier Transform Infrared Spectroscopy analysis of ConA and ConA/Ag/AgCl-NPs. **04**

Figure S3. Antibacterial activity of ConA against *S. aureus* and *P. aeruginosa*. **05**

Figure S4. Antibacterial activity of gentamicin against *S. aureus* and *P. aeruginosa*. **06**

Figure S5. Illustration of a 96-well plate used to determine the minimum inhibitory concentration (MIC) of gentamicin against *S. aureus*. **07**

Figure S6. Illustration of a 96-well plate used to determine the minimum inhibitory concentration (MIC) of gentamicin against *P. aeruginosa*. **08**

Figure S7. Illustration of a 96-well plate used to determine the minimum inhibitory concentration (MIC) of ConA/Ag/AgCl-NPs against *S. aureus*. **09**

Figure S8. Illustration of a 96-well plate used to determine the minimum inhibitory concentration (MIC) of ConA/Ag/AgCl-NPs against *P. aeruginosa*. **10**

Figure S9. Illustration of a 96-well plate used to assess the synergistic interaction between gentamicin and ConA protein against *P. aeruginosa*. **11**

Figure S10. Illustration of a 96-well plate used to assess the synergistic interaction between gentamicin and ConA protein against *S. aureus.* **12**

Figure S11. Illustration of a 96-well plate used to assess the synergistic interaction between gentamicin and *Sa*/Ag/AgCl-NPs protein against *S. aureus.* **13**

Figure S12. Illustration of a 96-well plate used to assess the synergistic interaction between gentamicin and *Sa*/Ag/AgCl-NPs protein against *P. aeruginosa*. **14**

Figure S13. Effect of ConA/Ag/AgCl-NPs on biofilm formation against *S. aureus* and *P. aeruginosa* at lower concentrations. **15**

Figure S14. Effect of ConA/Ag/AgCl-NPs on biofilm maintenance against *S. aureus* and *P. aeruginosa* at lower concentrations. **16**

Figure S15. The number of viable *S. aureus* cells (CFU/mL) in biofilms (A) and planktonic cell suspensions (B) after treatment with ConA/Ag/AgCl-NPs. **17**

Figure S16. The number of viable *P. aeruginosa* cells (CFU/mL) in biofilms (A) and planktonic cell suspensions (B) after treatment with ConA/Ag/AgCl-NPs. **18**

Figure S1. Characterization of ConA/Ag/AgCl-NPs. (**A**) Dynamic Light Scattering (DLS) size distribution of purified ConA/Ag/AgCl-NPs. (**B**) Zeta-potential measurement of ConA/Ag/AgCl-NPs. ConA/Ag/AgCl-NPs characterization.

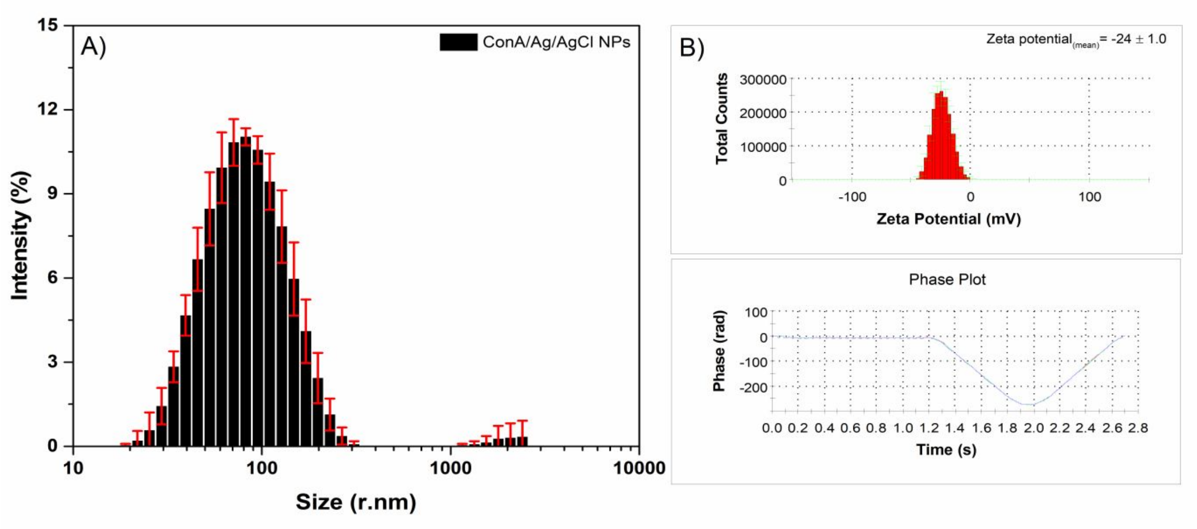

Figure S2. Fourier Transform Infrared Spectroscopy (FTIR) analysis of ConA (black line) and ConA/Ag/AgCl-NPs (red line).

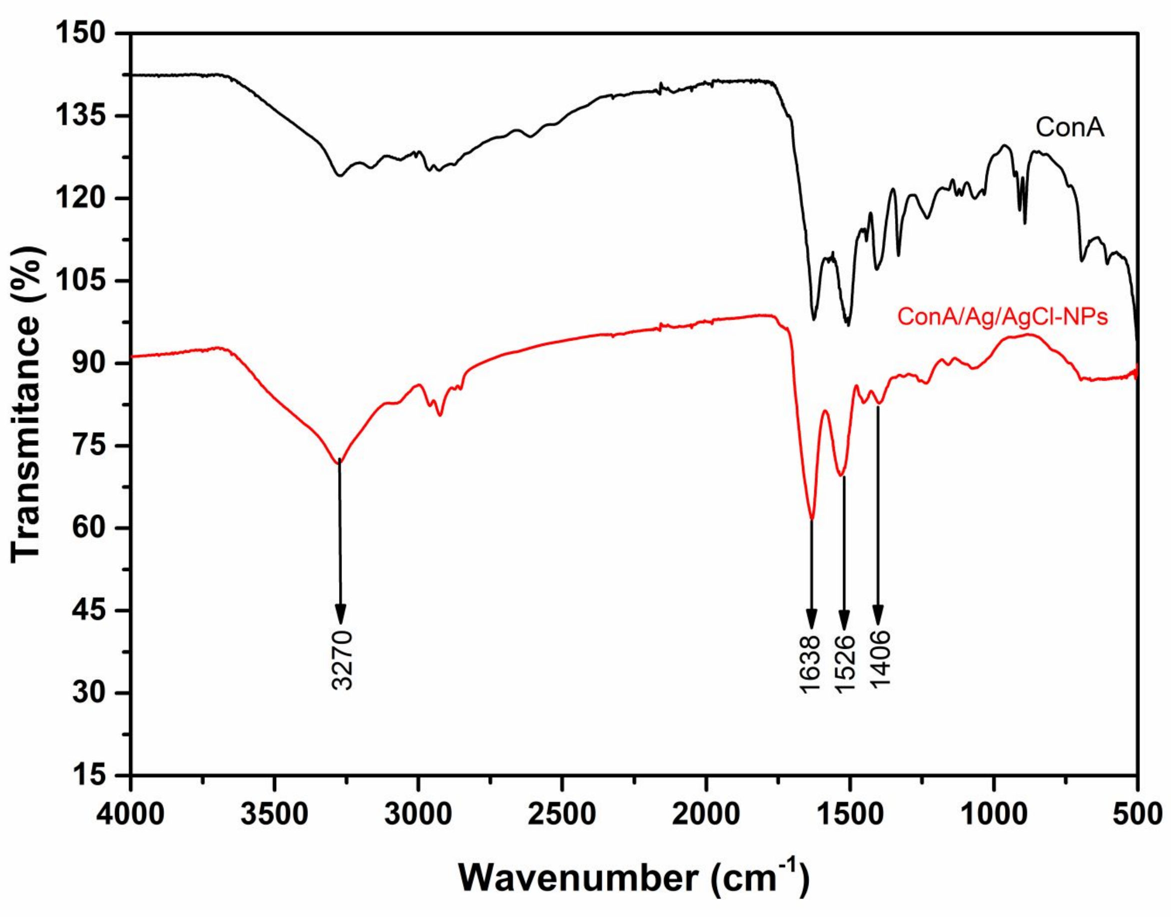

Figure S3. Antibacterial activity of ConA against *S. aureus* (**A**) and *P. aeruginosa* (**B**). Results were compared by the one-way ANOVA test and the statistical significance was determined by Tukey's test, with $p < 0.05$. The groups represented in "a" did not show statistically significant differences between them.

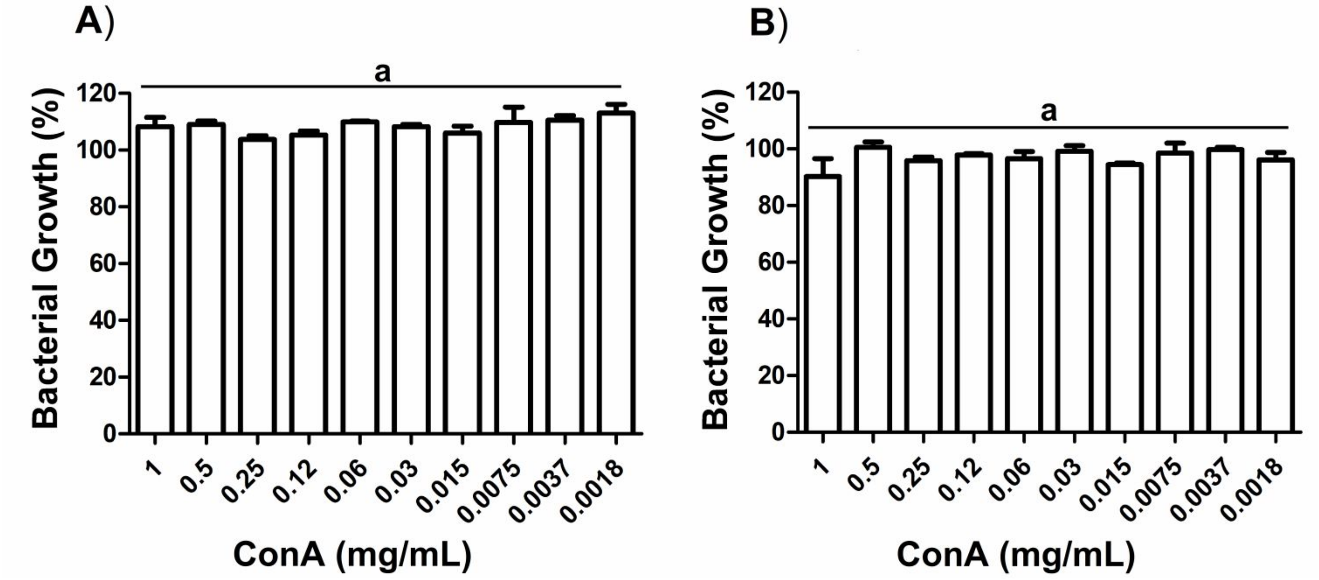

Figure S4. Antibacterial activity of gentamicin against *S. aureus* (**A**) and *P. aeruginosa* (**B**). Results were compared by the one-way ANOVA test and the statistical significance was determined by Tukey's test, with $p < 0.05$. The groups represented in "a" did not show statistically significant differences between them.

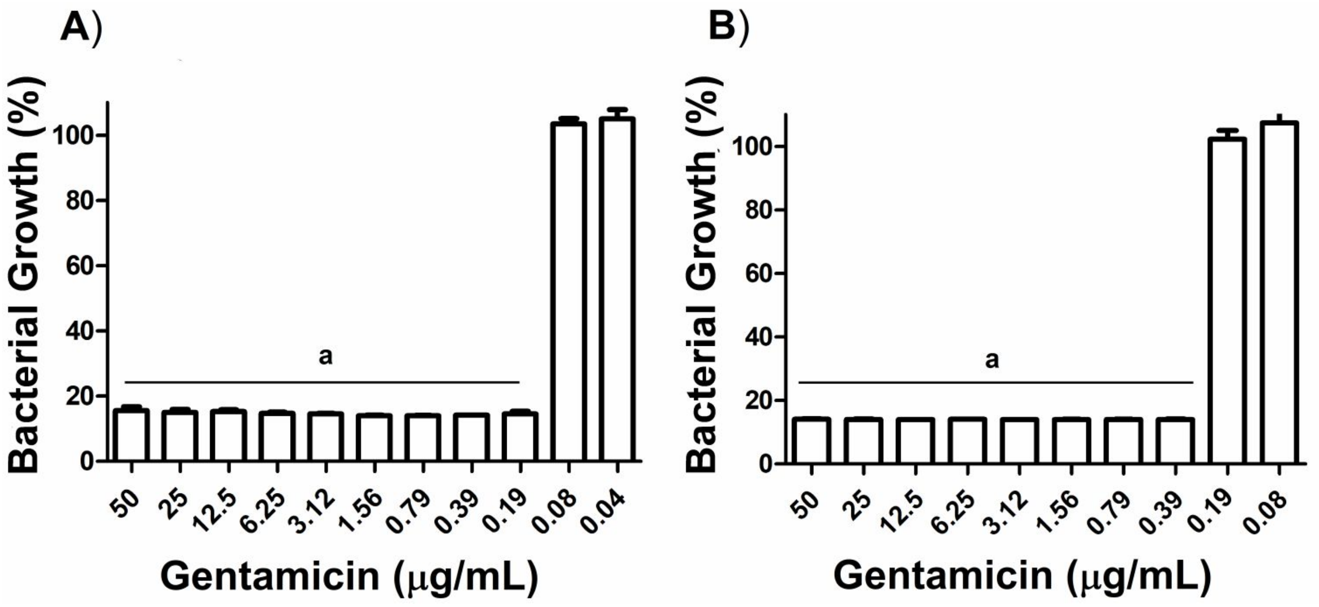

Figure S5. Illustration of a 96-well plate used to determine the minimum inhibitory concentration (MIC) of gentamicin against *S. aureus*. Yellow circles represent the culture medium control (negative control), pink circles indicate bacterial growth control (positive control), green circles correspond to the water control, and blue circles represent the bacterial growth rate (%) in wells treated with various gentamicin concentrations. A darker blue color indicates higher bacterial growth.

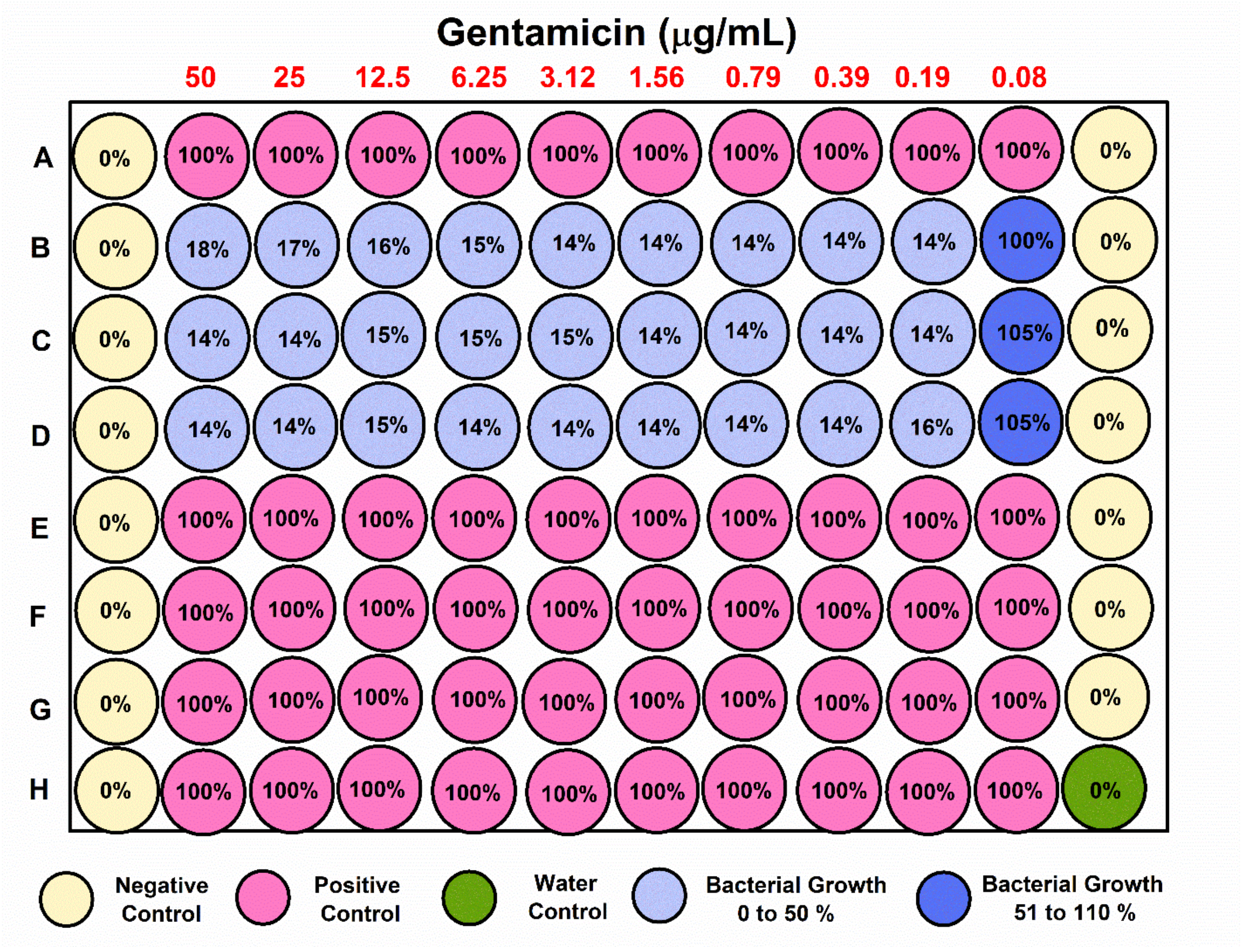

Figure S6. Illustration of a 96-well plate used to determine the minimum inhibitory concentration (MIC) of gentamicin against *P. aeruginosa*. Yellow circles represent the culture medium control (negative control), pink circles indicate bacterial growth control (positive control), green circles correspond to the water control, and blue circles represent the bacterial growth rate (%) in wells treated with various gentamicin concentrations. A darker blue color indicates higher bacterial growth.

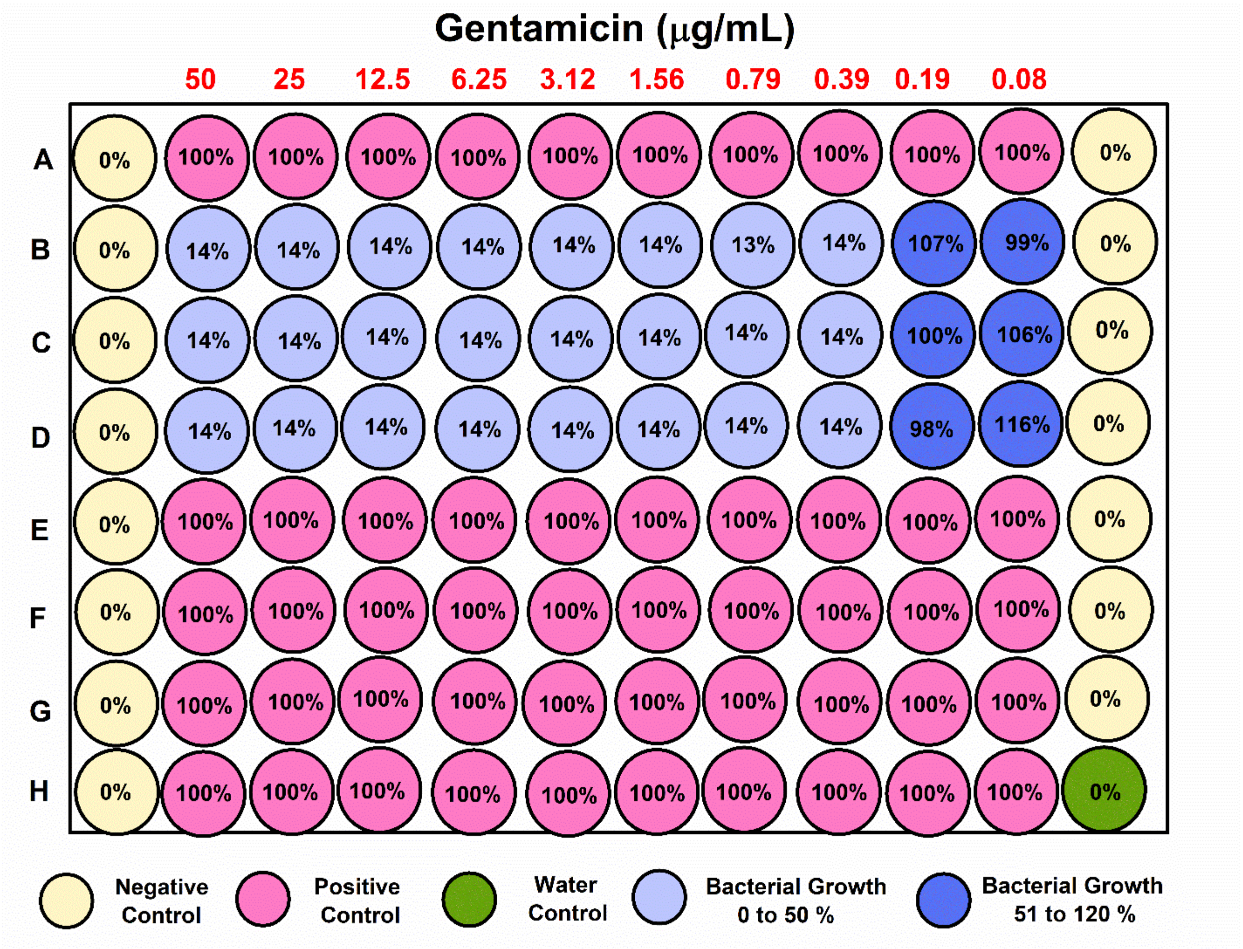

Figure S7. Illustration of a 96-well plate used to determine the minimum inhibitory concentration (MIC) of ConA/Ag/AgCl-NPs against *S. aureus*. Yellow circles represent the culture medium control (negative control), pink circles indicate bacterial growth control (positive control), green circles correspond to the water control, and blue circles represent the bacterial growth rate (%) in wells treated with various gentamicin concentrations. A darker blue color indicates higher bacterial growth.

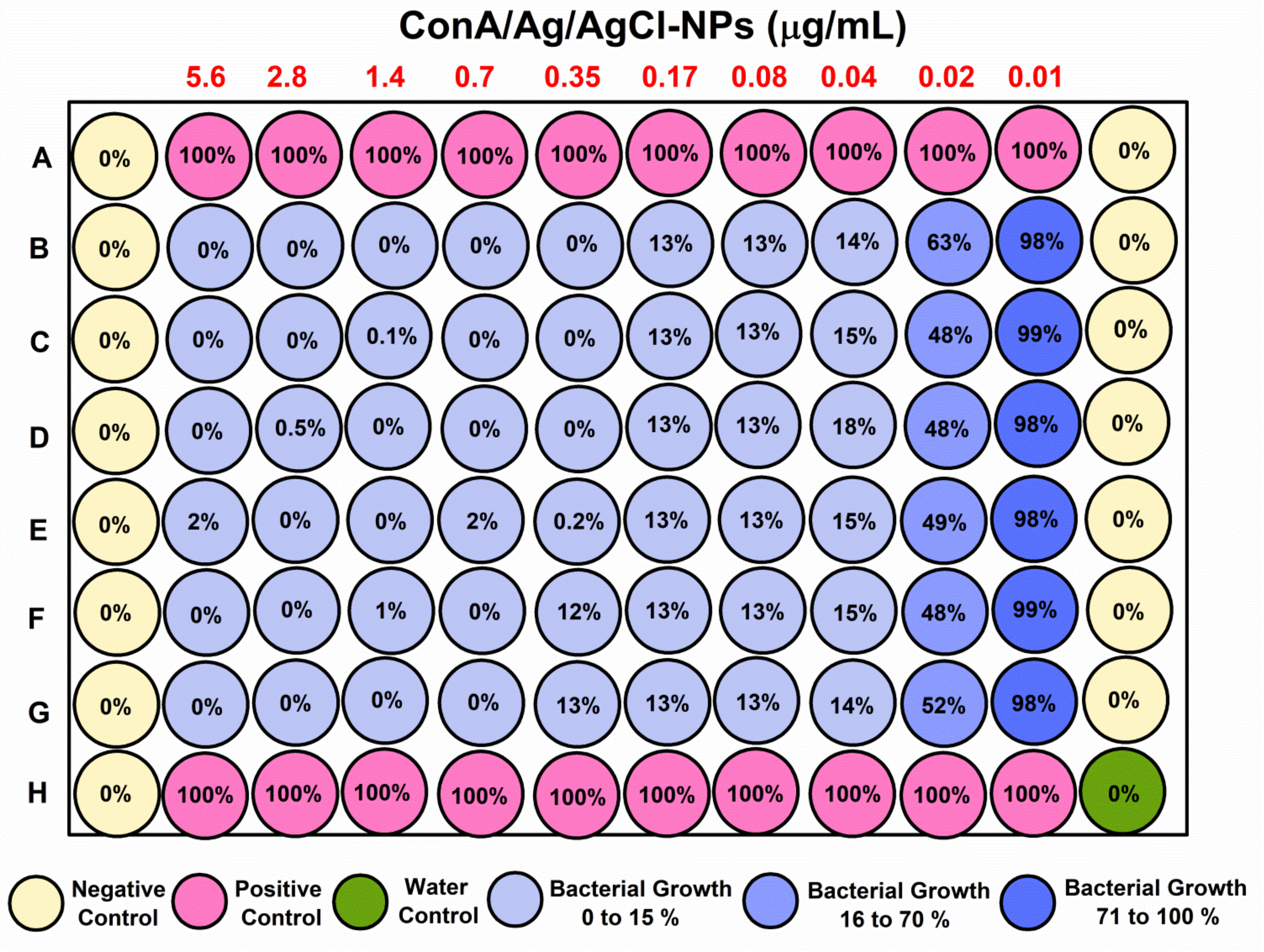

Figure S8. Illustration of a 96-well plate used to determine the minimum inhibitory concentration (MIC) of ConA/Ag/AgCl-NPs against *P. aeruginosa*. Yellow circles represent the culture medium control (negative control), pink circles indicate bacterial growth control (positive control), green circles correspond to the water control, and blue circles represent the bacterial growth rate (%) in wells treated with various gentamicin concentrations. A darker blue color indicates higher bacterial growth.

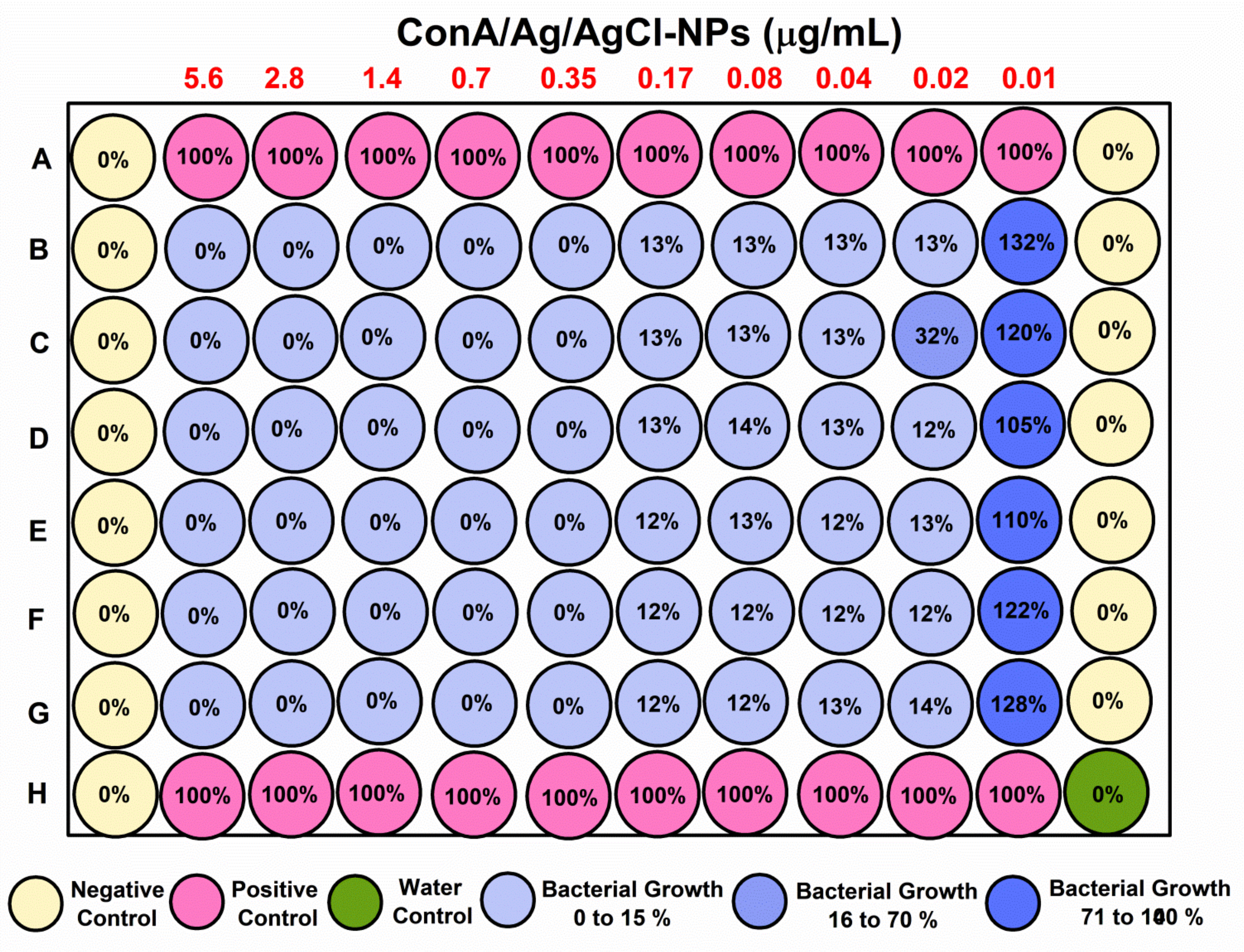

Figure S9. Illustration of a 96-well plate used to assess the synergistic interaction between gentamicin and ConA lectin against *P. aeruginosa*. Yellow circles represent concentrations that inhibited bacterial growth due to gentamicin alone (above its MIC of 0.39 µg/mL). Blue circles indicate concentrations where no synergistic, antagonistic, or additive effects were observed. The checkered white circle corresponds to the minimum inhibitory concentration that inhibits 90% of microbial growth (MIC_{90}) for gentamicin, and the green circle represents the bacterial growth control.

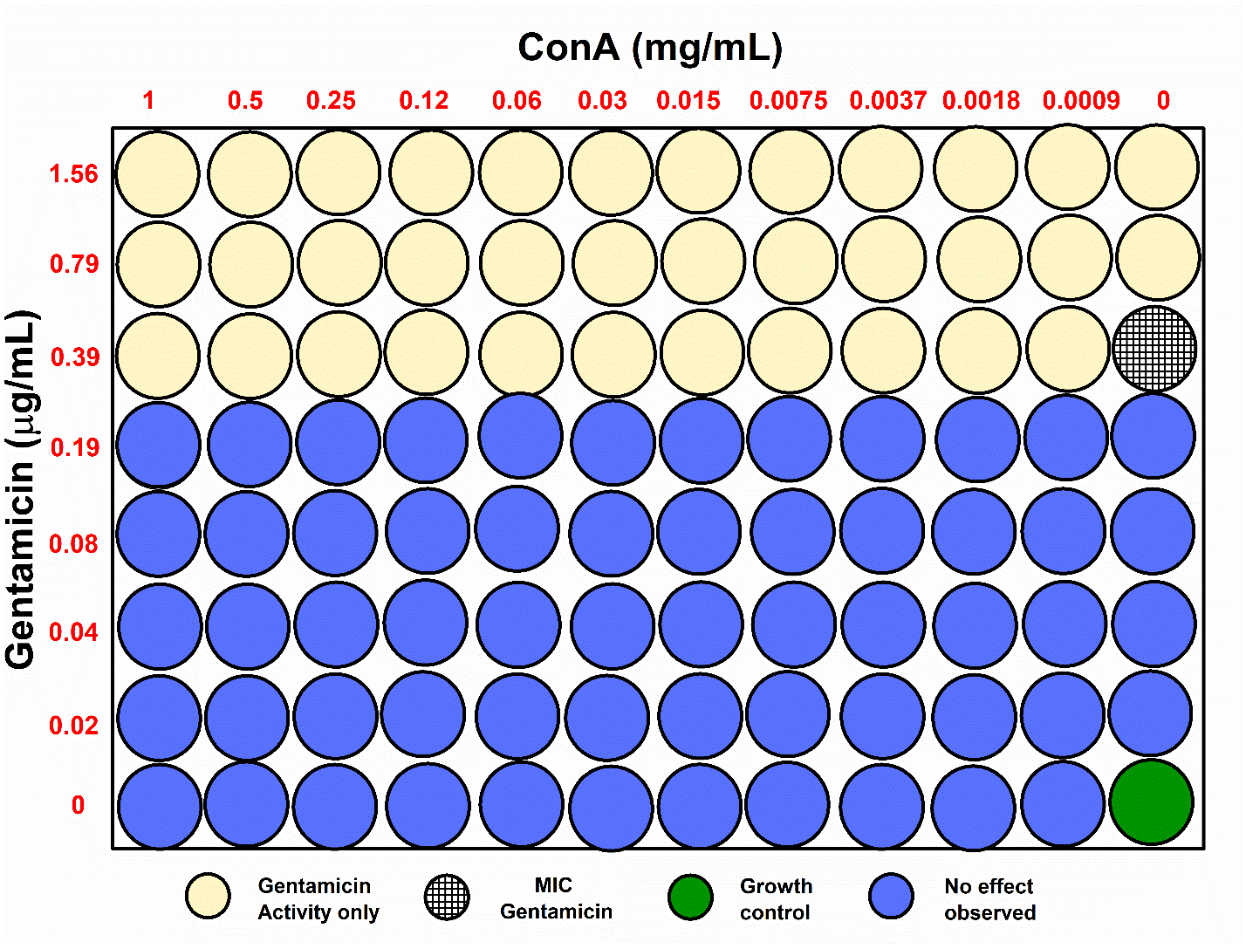

Figure S10. Illustration of a 96-well plate used to assess the synergistic interaction between gentamicin and ConA lectin against *S. aureus*. Yellow circles represent concentrations that inhibited bacterial growth due to gentamicin alone (above its MIC of 0.39 µg/mL). Blue circles indicate concentrations where no synergistic, antagonistic, or additive effects were observed. The checkered white circle corresponds to the minimum inhibitory concentration that inhibits 90% of microbial growth (MIC_{90}) for gentamicin, and the green circle represents the bacterial growth control.

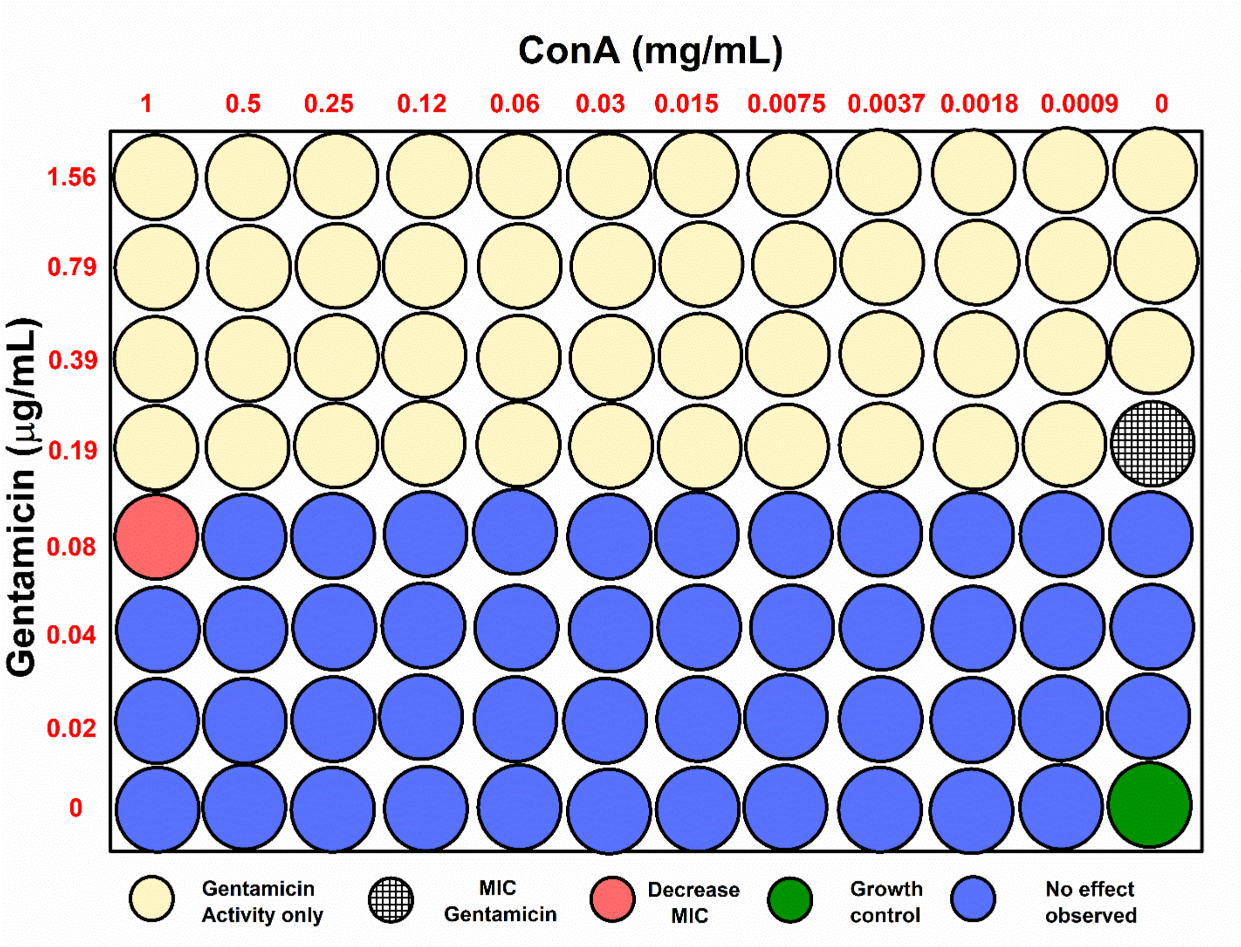

Figure S11. Illustration of a 96-well plate used to assess the synergistic interaction between gentamicin and *Sa*/Ag/AgCl-NPs against *S. aureus*. The yellow circles correspond to concentrations that had no effect with FIC index greater than 2.0. The orange circles correspond to concentrations that had an indifferent effect (FIC index > 1.0 to < 2.0), the cyan circles correspond to concentrations that had an additive effect (FIC index > 0.5 – 1.0) and the blue circles correspond to concentrations that did not show inhibition.

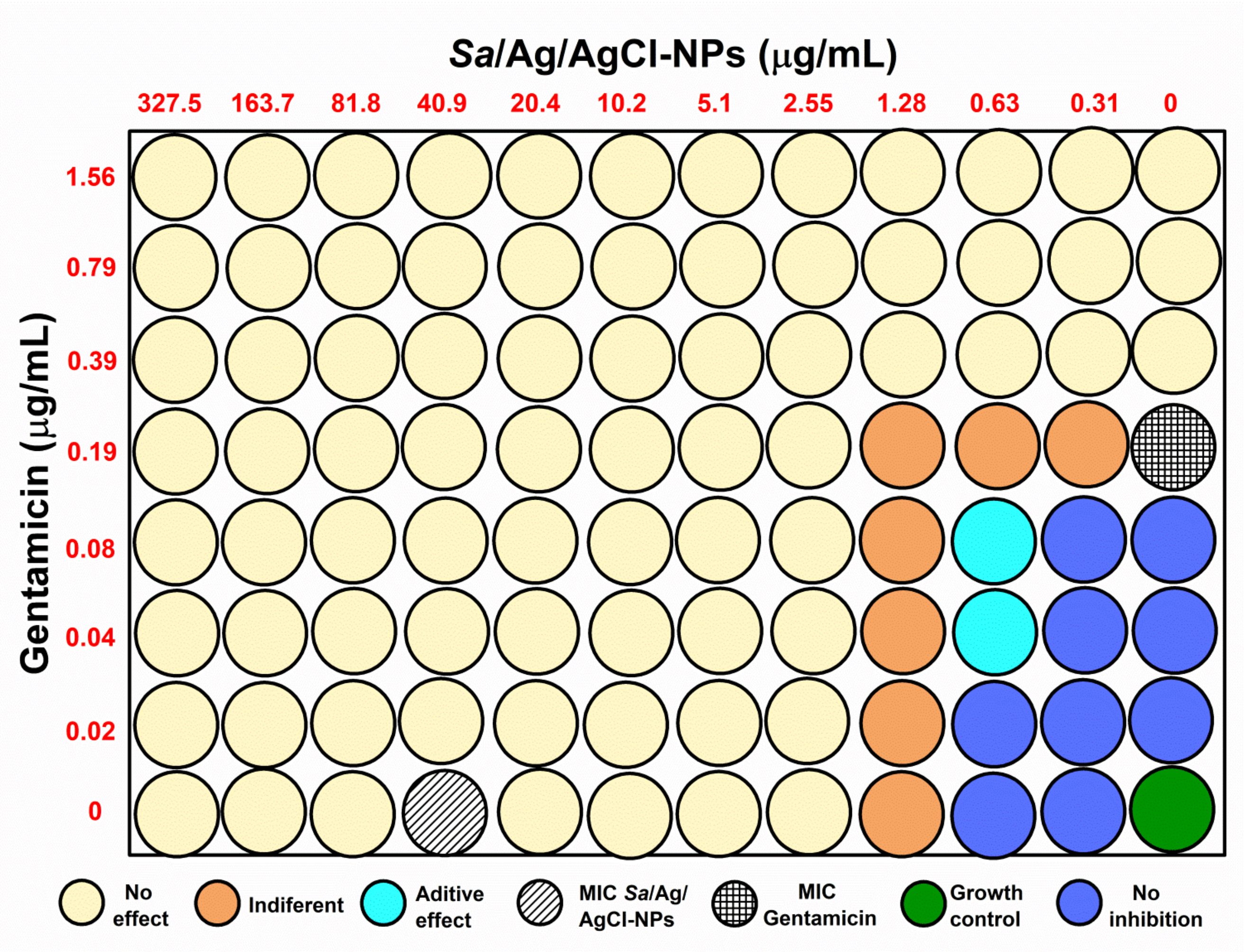

Figure S12. Illustration of a 96-well plate used to assess the synergistic interaction between gentamicin and *Sa*/Ag/AgCl-NPs against *P. aeruginosa*. The yellow circles correspond to concentrations that had no effect with FIC index greater than 2.0. The blue circles correspond to concentrations that did not show inhibition.

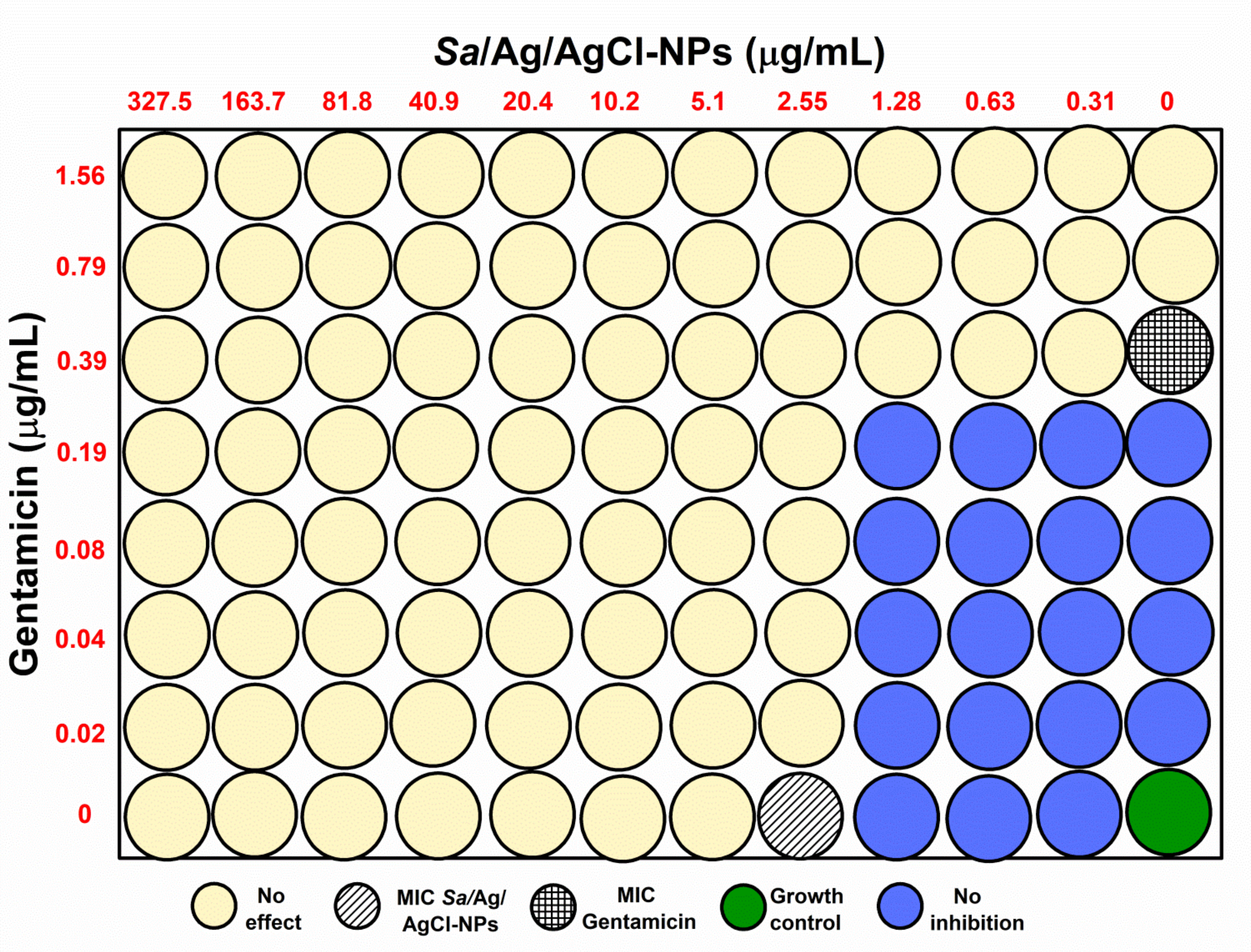

Figure S13. Effect of ConA/Ag/AgCl-NPs on biofilm formation against *S. aureus* (**A**) and *P. aeruginosa* (**B**) at lower concentrations. Results were compared by the one-way ANOVA test and the statistical significance was determined by Tukey's test, with $p < 0.05$. The groups represented in "a" did not show statistically significant differences between them.

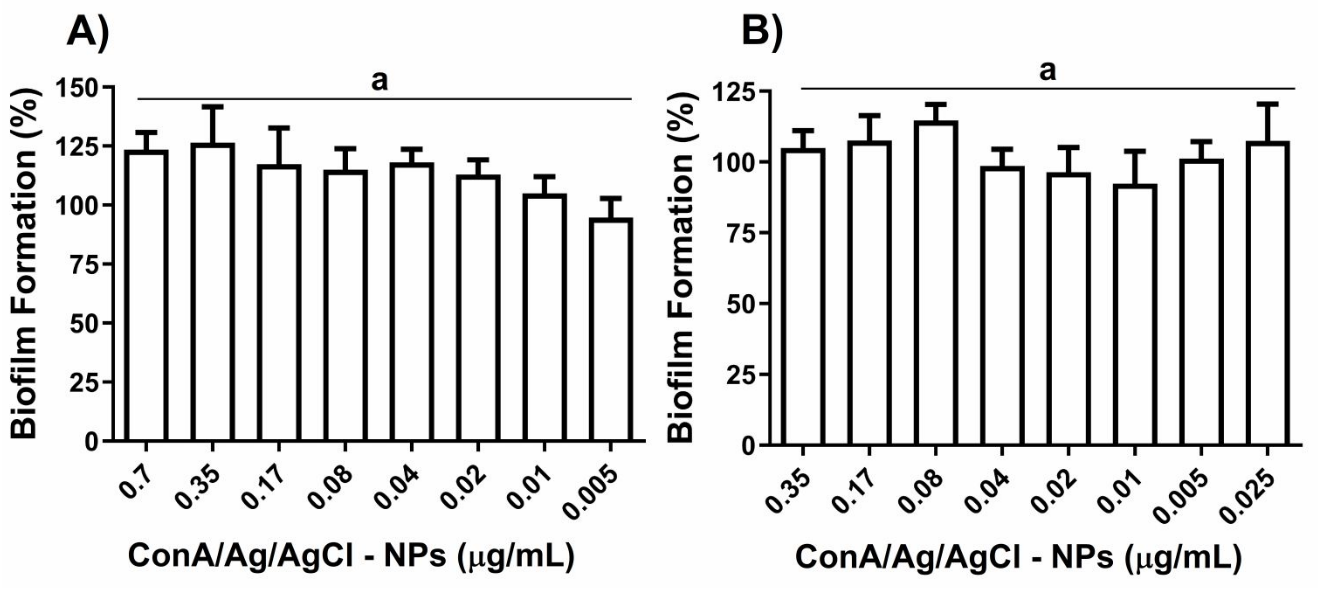

Figure S14. Effect of ConA/Ag/AgCl-NPs on biofilm maintenance against *S. aureus* (**A**) and *P. aeruginosa* (**B**) at lower concentrations. Results were compared by the one-way ANOVA test and the statistical significance was determined by Tukey's test, with $p < 0.05$. The groups represented in "a" did not show statistically significant differences between them.

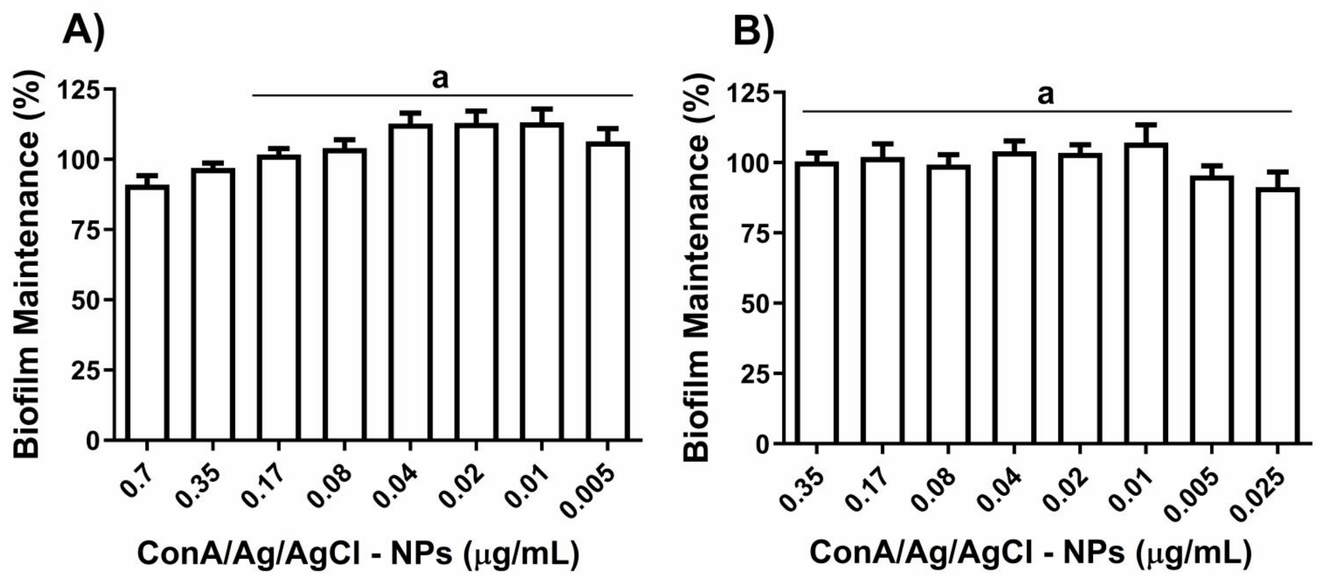

Figure S15. The number of viable *S. aureus* cells (CFU/mL) in biofilms (**A**) and planktonic cell suspensions (**B**) after treatment with ConA/Ag/AgCl-NPs. The groups represented in “a” did not show statistically significant differences between them, and the groups represented in "b" show statistically significant differences.

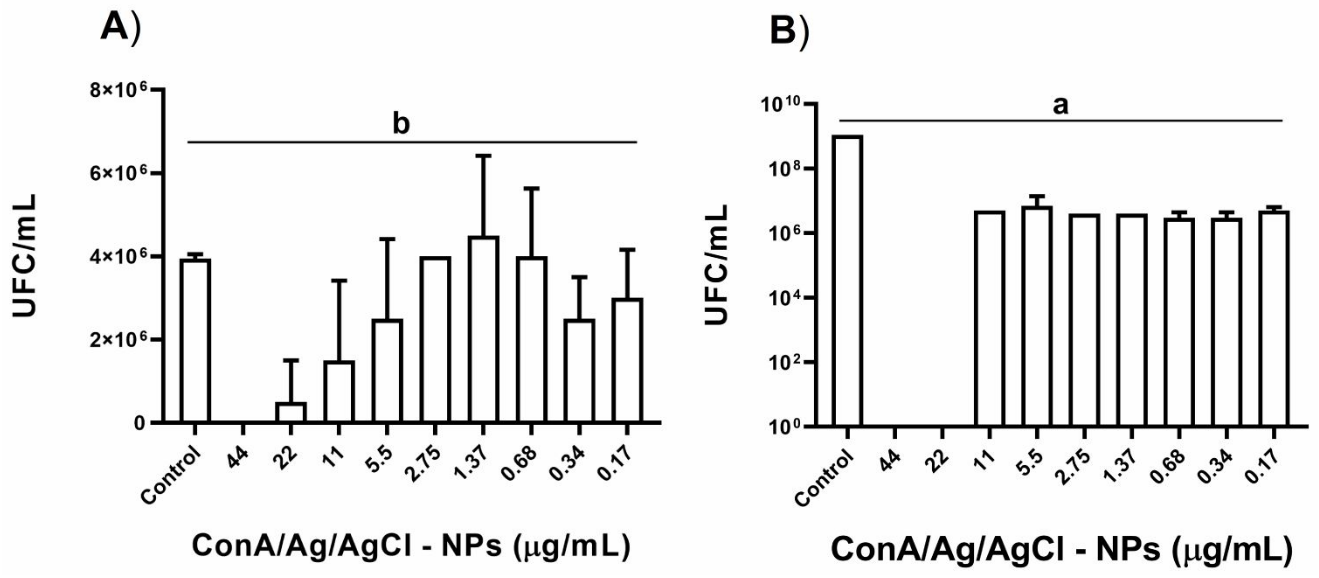

Figure S16. The number of viable *P. aeruginosa* cells (CFU/mL) in biofilms (**A**) and planktonic cell suspensions (**B**) after treatment with ConA/Ag/AgCl-NPs. The groups represented in "b" show statistically significant differences.

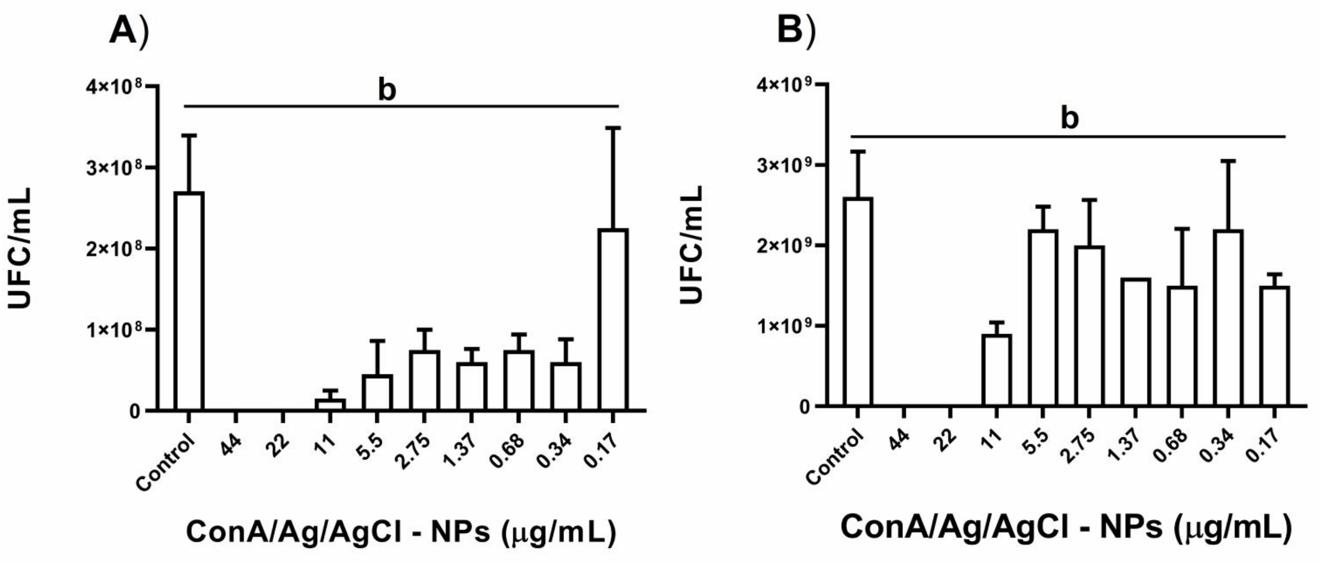

Supplement: Supplementary file 1 [file ao5c03081_si_001.pdf]
